# Intron retention is regulated by altered MeCP2-mediated splicing factor recruitment

**DOI:** 10.1038/ncomms15134

**Published:** 2017-05-08

**Authors:** Justin J. -L. Wong, Dadi Gao, Trung V. Nguyen, Chau-To Kwok, Michelle van Geldermalsen, Rob Middleton, Natalia Pinello, Annora Thoeng, Rajini Nagarajah, Jeff Holst, William Ritchie, John E. J. Rasko

**Affiliations:** 1 Gene & Stem Cell Therapy Program, Centenary Institute, University of Sydney, Camperdown, New South Wales 2050, Australia; 2 Gene Regulation in Cancer Laboratory, Centenary Institute, University of Sydney, Camperdown, New South Wales 2050, Australia; 3 Sydney Medical School, University of Sydney, Sydney, New South Wales 2006, Australia; 4 Bioinformatics Laboratory, Centenary Institute, University of Sydney, Camperdown, New South Wales 2050, Australia; 5 Origins of Cancer Program, Centenary Institute, University of Sydney, Camperdown, New South Wales 2050, Australia; 6 CNRS, UMR 5203, 34094 Montpellier, France; 7 Cell and Molecular Therapies, Royal Prince Alfred Hospital, Camperdown, New South Wales 2050, Australia

## Abstract

While intron retention (IR) is considered a widely conserved and distinct mechanism of gene expression control, its regulation is poorly understood. Here we show that DNA methylation directly regulates IR. We also find reduced occupancy of MeCP2 near the splice junctions of retained introns, mirroring the reduced DNA methylation at these sites. Accordingly, MeCP2 depletion in tissues and cells enhances IR. By analysing the MeCP2 interactome using mass spectrometry and RNA co-precipitation, we demonstrate that decreased MeCP2 binding near splice junctions facilitates IR via reduced recruitment of splicing factors, including Tra2b, and increased RNA polymerase II stalling. These results suggest an association between IR and a slower rate of transcription elongation, which reflects inefficient splicing factor recruitment. In summary, our results reinforce the interdependency between alternative splicing involving IR and epigenetic controls of gene expression.

## Abstract (web summary)

Intron retention is a conserved mechanism that controls gene expression but its regulation is poorly understood. Here, the authors provide evidence that DNA methylation regulates intron retention and find reduced MeCP2 occupancy and splicing factor recruitment near affected splice junctions.

Alternative splicing (AS) is essential for diverse physiological functions including cell division, differentiation, stress response and lineage specification^1^,^2^,^3^,^4^,^5^. About one-fifth of human diseases are caused by disrupted splicing^6^, making normal AS a vital regulatory mechanism in humans. Accordingly, the search for mechanisms underlying AS has been one of the most active research areas in molecular and cellular biology.

One mode of AS termed intron retention (IR) occurs when an intron is not excised by the spliceosome, and is preserved in the final mature messenger RNA (mRNA). Intron-retaining transcripts typically contain premature termination codons, resulting in their removal by surveillance mechanisms including nonsense-mediated decay (NMD). Whilst AS generates proteomic complexity from most genes, IR is a distinctive mechanism that reduces gene expression, which in turn regulates cellular differentiation^1^,^2^,^7^,^8^. Following our initial report that numerous introns are constitutively retained to induce mRNA degradation via NMD during normal granulopoiesis^2^, the prevalence of IR in ∼40 cell and tissue types, and its conservation across 11 vertebrate species has been established^9^. In general, the molecular mechanisms that regulate IR have not been thoroughly examined. However, in one well-characterized example, the H3.3K36me3 reader, BS69, binds to a U5 associated splicing factor, EFTUD2, to antagonize splicing activity, leading to increased IR (ref. 10). Of all IR events identified in that study, only one-fifth were BS69-dependent^10^. Thus other as yet undefined mechanisms exist to regulate IR.

Much is known about the mechanisms that regulate other modes of AS including exon skipping and inclusion, but their relevance to the control of IR remains uncertain. RNA Poll II stalling, which improves splice signal recognition is integral to the ‘kinetic' model of AS that involves epigenetic changes^11^. For example, altered DNA methylation levels and downstream binding of transcription factors as well as regulatory proteins such as CTCF and MeCP2 induce RNA Pol II stalling to promote alternative exon skipping and inclusion^12^,^13^. RNA Pol II stalling has been observed near splice junctions of retained introns but it is unclear if it directly regulates IR or whether other cis- and/or trans- regulatory factors initiate the process^9^. Also, lower DNA methylation levels near splice junctions have been associated with increased IR in normal human breast tissues^14^. This latter finding raises the intriguing possibility that DNA methylation might regulate IR by altering RNA Pol II processivity and splice site recognition according to the ‘kinetic' model.

Alternatively, RNA Pol II stalling near splice junctions of retained introns could occur consequent to inefficient splicing factor recruitment^15^,^16^. This ‘recruitment' model of AS has been shown to function in the case of exon skipping, which is also associated with DNA methylation changes^17^,^18^. Taken together changes in DNA methylation and RNA Pol II elongation are both factors that are known to be associated with IR. However, it is currently unknown whether DNA methylation directly controls IR in the same manner by which it controls exon skipping and inclusion. Any functional connection between DNA methylation and the ‘kinetic' and ‘recruitment' models in the regulation of IR has been an open question to date.

In this study, we sought to determine whether IR is regulated by spliceosomal and DNA methylation changes and how they are linked. We show that lower levels of DNA methylation near 5′ and 3′ splice junctions are significantly associated with increased IR in a myriad of primary cells and cell lines. Importantly, both chemical and genetic inhibition of DNA methylation promoted IR in mouse embryonic stem cells, mouse B cells, and in both mouse and human cell lines. We show reduced MeCP2 binding near 5′ and 3′ splice junctions of retained introns in mouse granulocytes and promyelocytes, which corresponds with the physiological decrease in DNA methylation levels. Further, knockdown or knockout of MeCP2 in primary cells and cell lines led to increased IR. To confirm that MeCP2 occupancy influences the efficiency of splicing, we show that reduced MeCP2 occupancy is associated with RNA Pol II stalling. Using immunoprecipitation followed by mass spectrometry (MS), we further show that many splicing factors are associated with MeCP2 including the Srsf family members and Tra2b. Consistent with inefficient splicing factor recruitment as reflected by RNA Pol II stalling, we demonstrate reduced binding of the splicing factor Tra2b to RNA transcripts containing retained introns along with reduced binding of MeCP2 to these transcripts. Our data link the two models of splicing by demonstrating a direct causative role of decreased DNA methylation in promoting IR via reduced MeCP2 occupancy, which in turn perturbs the recruitment of splicing factors such as Tra2b, leading to RNA Pol II stalling.

## Results

### IR correlates with high CpG density and low gene expression

We performed fluorescence-activated cell sorting (FACS) of primary mouse promyelocytes and granulocytes isolated from mouse bone marrow according to our established protocol (Supplementary Fig. 1a,b)^2^. Our previous conservative algorithm to detect IR identified 121 introns from 86 transcripts that show differential IR in granulocytes compared to promyelocytes^2^. We have now refined our previous IRFinder algorithm to better estimate IR from sequencing reads and to include more introns in our analysis. This is achieved mainly by removing biases such as low mappability and overlapping features but also using split reads as a measure of correct splicing surrounding IR (Methods section). This method allows for a more sensitive detection of IR and of differential IR. Reanalysis of our data estimates 737 differentially retained introns (*P*<0.05, Audic and Claverie test) between the two cell types out of a total of 3,200 and 3,888 introns retained (IR level ≥10%) in promyelocytes and granulocytes, respectively. Of these 737 differentially retained introns, substantially more introns (687/737, 93.22%) showed higher levels of retention in granulocytes (Supplementary Fig. 1c); consistent with our previous report^2^. Gene ontology analysis (Supplementary Fig. 1d) again reinforced significant representation of features associated with granulopoiesis and granulocyte maintenance, including nuclear lamina^2^,^19^ as well as metabolic and catabolic processes involving different types of lipids^20^,^21^,^22^.

Retained introns were significantly shorter in nucleotide length than non-retained introns (*P*<1.00e-16, *t*-test, Supplementary Fig. 1e). Retained introns were also significantly associated with higher intronic GC content and CpG density (*P*<0.05, *t*-test, Supplementary Fig. 1f,g), and higher CpG density near their flanking exon–intron boundaries (*P*<0.05, *t*-test, Supplementary Fig. 1g).

Consistent with previous reports^2^,^9^ we also found that higher IR levels were associated with lower expression of coding mRNA in promyelocytes and granulocytes (Supplementary Fig. 1h,i). These results indicate downregulation of gene expression via IR-triggered NMD or sequestering of intron-detained transcripts in the nucleus as previously reported^1^,^2^,^8^,^9^.

Notably, the average distance between two genes with a retained intron was significantly shorter than the average distance between two randomly selected genes (*P*<1.00e-16, *t*-test, Supplementary Fig. 2a). Moreover, introns are frequently retained in the same gene and these are often adjacent to each other (*P*<1.00e-16, *t*-test, Supplementary Fig. 2b). This result not only reflects that multiple tandem introns are being retained in a given transcript but that IR may concomitantly affect many RNA transcripts encoded by neighbouring genes within the same chromosomal region.

### Reduced DNA methylation at splice junctions marks IR

**Figure 1:**
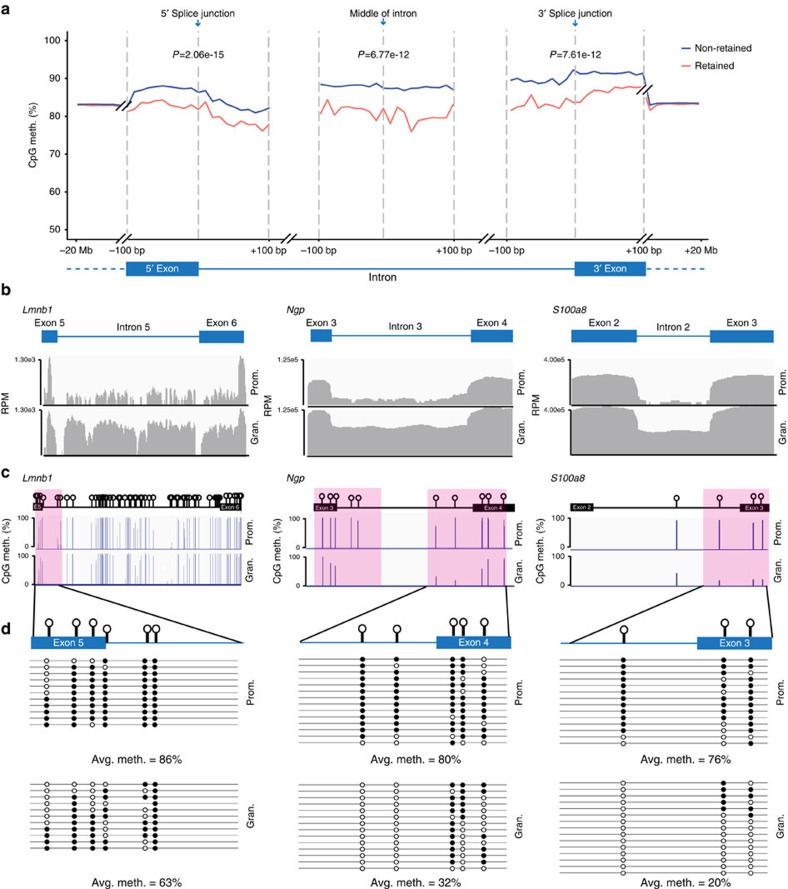
IR in myeloid cells is associated with lower DNA methylation levels near splice junctions and within retained introns. (**a**) Average CpG methylation (CpG meth.) levels spanning ±100 bp from the 5′ and 3′ splice junctions, and from the middle of introns, in the DNA encoding non-retained (blue) and retained (red) introns in promyelocytes and granulocytes combined. Data were extended to −20 Mb and +20 Mb from the 5′ and 3′ splice junction respectively. *t*-test was used to determine significance at *P*<0.05. (**b**) Examples from the IGV genome browser comparing IR levels in *Lmnb1* intron 5, *Ngp* intron 3 and *S100a8* intron 2 in promyelocytes (Prom.) and granulocytes (Gran.). (**c**) Average DNA methylation level at each CpG across *Lmnb1* intron 5, *Ngp* intron 3 and *S100a8* intron 2 and their flanking exons in promyelocytes and granulocytes as assessed using whole-genome bisulfite sequencing. A ±100 bp region from each splice junction, with reduced DNA methylation in granulocytes, is shaded in pink. (**d**) Confirmation of the results in **c** using clonal bisulfite sequencing. Each row represents a single-cloned PCR amplicon aligned to the exon–intron map shown above. Lollipops on exon–intron map indicate positions of CpG sites. Circles denote methylated (black) and unmethylated (white) CpGs. NS, not significant; Avg. meth., average DNA methylation levels.

DNA methylation occurs predominantly at CpG dinucleotides in mammals^23^. Changes in DNA methylation levels have established roles in the regulation of other modes of splicing including exon-inclusion and skipping^12^,^13^,^18^,^24^. Therefore, the strong association between increased CpG density and IR (Supplementary Fig. 1g) prompted us to investigate whether DNA methylation also regulates IR. In order to determine the association between DNA methylation and IR, we performed whole-genome bisulfite sequencing (WGBS) on genomic DNA from promyelocytes and granulocytes. A total of 560 million and 633 million paired-end reads were aligned to the mouse genome (mm10) for promyelocytes and granulocytes, respectively. To measure the methylation level of each CpG in the genome, the beta value was calculated for each CpG (Methods section). Sharp transitions in DNA methylation patterns are known to mark splice junctions, suggesting that these signals may be crucial for the recognition of exons and introns^25^. We measured the levels of DNA methylation within ±100 bp of 5′ or 3′ splice junctions of all introns in promyelocytes and granulocytes. Significantly lower DNA methylation levels exist near both 5′ and 3′ junctions of retained introns compared to non-retained introns (*P*=2.06e-15 and 7.61e-12, *t*-test, respectively; Fig. 1a). This result indicates that reduced DNA methylation marks the junctions of introns that are more likely to be retained (Fig. 1a). Significantly lower DNA methylation levels also occurred within a 200-bp region spanning the middle of retained introns (*P*=6.77e-12, *t*-test, Fig. 1a).

Of 737 differentially retained introns in granulocytes and promyelocytes, 125 (16.9%) showed reduced DNA methylation levels by ≥10% near splice junctions or within introns when IR levels increased in either cell type. This result indicates that DNA methylation-associated IR events may occur at a similar frequency as BS69-associated IR events^10^. Importantly, IR-associated DNA methylation changes affect numerous genes, including those relevant to granulopoiesis and granulocyte functions: *Lmnb1* (refs 2, 19), *Ngp* (ref. 26), *S100a8* (ref. 27), *Mmp8* (ref. 28), *Coro1a* (ref. 29), *S100a9* (ref. 27), *Gadd45a* (ref. 30) and *Atf4* (ref. 31; Fig. 1b,c and Supplementary Fig. 3). Clonal bisulfite sequencing confirmed lower DNA methylation levels are associated with increased IR levels for *Lmnb1* intron 5, *Ngp* intron 3 and *S100a8* intron 2 in granulocytes (Fig. 1d).

### Reduced DNA methylation associated with IR is widespread

**Figure 2:**
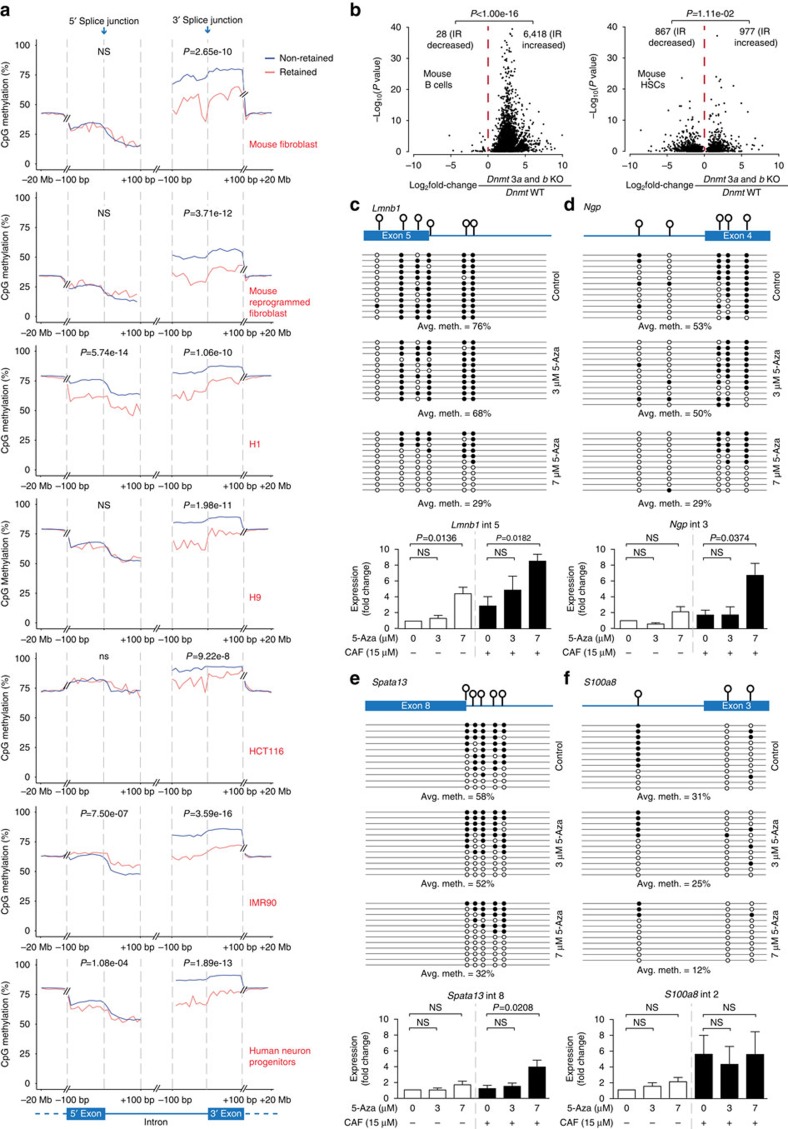
IR increases with reduced DNA methylation and increases following DNA methylation inhibition. (**a**) Average CpG methylation levels spanning ±100 bp from the 5′ and 3′ splice junctions in the DNA encoding non-retained (blue) and retained (red) introns in primary mouse fibroblasts, mouse reprogrammed fibroblasts, human cell lines (H1, H9, HCT116 and IMR90) and primary human neuron progenitors. Data were extended to −20 Mb and +20 Mb from the 5′ and 3′ splice junctions, respectively. Paired *t*-tests were used to determine significance at *P*<0.05. (**b**) Association between the IR ratio fold changes and their significance following double knockout of *Dnmt3a* and *3b* in primary mouse B cells and HSCs. Binomial test was used to determine the significance of bias between increased and decreased IR at *P*<0.05. (**c**–**f**) Upper panel: clonal bisulfite sequencing displaying DNA methylation changes near splice junctions of *Lmnb1* intron 5, *Ngp* intron 3, *Spata13* intron 8 and *S100a8* intron 2 in MPRO cells following treatment with 3 or 7 μM 5-aza-2′deoxycytidine (5-Aza). Each row represents a single-cloned PCR amplicon aligned to the exon–intron map shown. Each lollipop on the map represents a CpG site. Black and white circles denote methylated and unmethylated CpGs respectively. Lower panel: IR levels determined by qRT-PCR for specific introns indicated in MPRO cells, with and without exposure to 5-Aza at 3 or 7 μM, and in the absence (–) or presence (+) of NMD inhibition via caffeine (CAF) treatment. Two-tailed *t*-test was used to determine significance at *P*<0.05. Bars display mean±s.e.m. Independent qRT-PCR experiments were performed three times in triplicate (*n*=3). NS, not significant; Avg. meth., average DNA methylation levels.

We next set out to determine the extent of IR events that are marked by reduced DNA methylation near splice junctions. We analysed publicly available matched RNA-seq and methylome data from normal and cancer cells, including mouse primary and reprogrammed fibroblasts, human embryonic stem cell lines (H1 and H9), a human lung fibroblast cell line (IMR90), a human colon cancer cell line (HCT116) and human neuron progenitor cells (see Supplementary Table 1 for details). Consistent with our findings in myeloid cells, significantly lower DNA methylation levels were observed near 3′ splice junctions flanking retained compared to non-retained introns in all cell types examined (*P*<9.22e-8 in all cases, *t*-test, Fig. 2a). Lower DNA methylation levels near 5′ splice junctions were significantly associated with retained introns in the H1 cell line and human neuron progenitors, but not in other cells (Fig. 2a). Lower levels of DNA methylation were also present within the body of retained introns compared to non-retained introns in all cell types (*P*<1.33e-5 in all cases, paired *t*-test, Supplementary Fig. 4a). Overall, lower DNA methylation levels near 3′ splice junctions and within introns consistently distinguished retained from non-retained introns in all cell types examined, demonstrating conservation of this phenomenon in human and mouse biology.

### Inhibition of DNA methylation increases intron retention

To determine whether DNA methylation changes can directly alter IR levels, we reanalysed RNA-seq data of cells with reduced DNA methylation, achieved via 5-Aza-2′-deoxycytidine (5-Aza) treatment or *Dnmt*-knockout (*Dnmt*-KO; see Supplementary Table 1 for details). *DNMT1* is the maintenance DNA methyltransferase while both *DNMT3A* and *3B* direct *de novo* DNA methylation within coding regions, and their binding patterns exhibit a high degree of overlap^32^. We identified 6,446, 1,844 and 20 differentially retained introns in *Dnmt3a* and *3b*-double-KO mouse B cells, *Dnmt*3a and *3b-*double-KO haemopoietic stem cells (HSCs) and 5-Aza-treated leukaemic OCI-AML3 cells compared to respective wild type and untreated cells. The number of introns exhibiting increased IR levels (*Dnmt3a* and *3b*-double-KO mouse B cells: 6418/6446, 99.57%; *Dnmt3a* and *3b*- double-KO HSCs: 977/1844, 52.98%; 5-Aza-treated OCI-AML3: 15/20, 75%) was significantly more than introns with decreased IR levels in all three cell types (*P*<0.05, binomial test, Fig. 2b and Supplementary Fig. 4b). These results indicate that inhibition of DNA methylation is more likely to promote than inhibit IR. Notably, a lower percentage of introns with increased IR levels was observed in *DNMT3B* and hypomorphic *DNMT1* double-KO HCT116 cells (224/613, 37%) compared to control (*P*<0.05 binomial test) (Supplementary Fig. 4b). This discrepancy may be due either to incomplete *DNMT1* loss or the compensatory presence of *DNMT3A*.

We also treated the mouse promyelocyte cell line, MPRO, with 5-Aza, to determine the changes in the levels of nine intron-retaining transcripts following DNA demethylation. These intron-retaining transcripts were selected from those showing increased IR and concomitantly reduced DNA methylation levels in granulocytes compared to promyelocytes (Fig. 1b–d and Supplementary Fig. 3). Since IR typically leads to NMD, we also inhibited NMD using caffeine treatment to measure the changes in IR when RNA degradation is limited. In three IR-affected genes, decreased DNA methylation levels near 5′ or 3′ splice sites in the DNA were associated with significant increases in IR levels (Fig. 2c–e). For *Lmnb1*, the IR levels were significantly increased by >four-fold with (0 μM Aza: 7 μM Aza +caffeine, *P*=0.0182, *t*-test) and without NMD inhibition (0 μM Aza: 7 μM Aza, *P*=0.0136, *t*-test; Fig. 2c). For *Ngp* and *Spata13*, significant increases in IR levels by >6 (*P*=0.0374, *t*-test) and >4-fold (*P*=0.0208, *t*-test), respectively, were achieved following NMD inhibition (Fig. 2d,e). For the remaining six transcripts, IR levels did not change following 5-Aza treatments (Fig. 2f and Supplementary Fig. 4c–g). For five genes (*Mmp8*, *S100a9*, *Gart*, *Gadd45a* and *Atf4*), these results may be explained by the lack of changes in DNA methylation levels near 5′ or 3′ splice sites despite 5-Aza exposure (Supplementary Fig. 4c–g). For *S100a8*, constitutive DNA methylation levels were low in untreated MPRO cells (average DNA methylation of 31% compared to 76% in primary promyelocytes), and further demethylation by 5-Aza failed to alter IR levels (Fig. 2f). Notably, treatment with a lower concentration of 3 μM 5-Aza- was insufficient to cause substantial decrease in DNA methylation levels near 5′ or 3′ splice sites of all 10 introns (Fig. 2c–f and Supplementary Fig. 4c–g). We conclude that because no significant changes in DNA methylation levels were detected in cells treated with lower dose 5-Aza compared to untreated controls, there was no significant difference produced in IR levels. Overall, our data indicate that when DNA methylation levels near 5′ or 3′ splice sites flanking introns are substantially reduced, there is an increase in IR levels.

### IR splice sites show reduced MeCP2 binding

**Figure 3:**
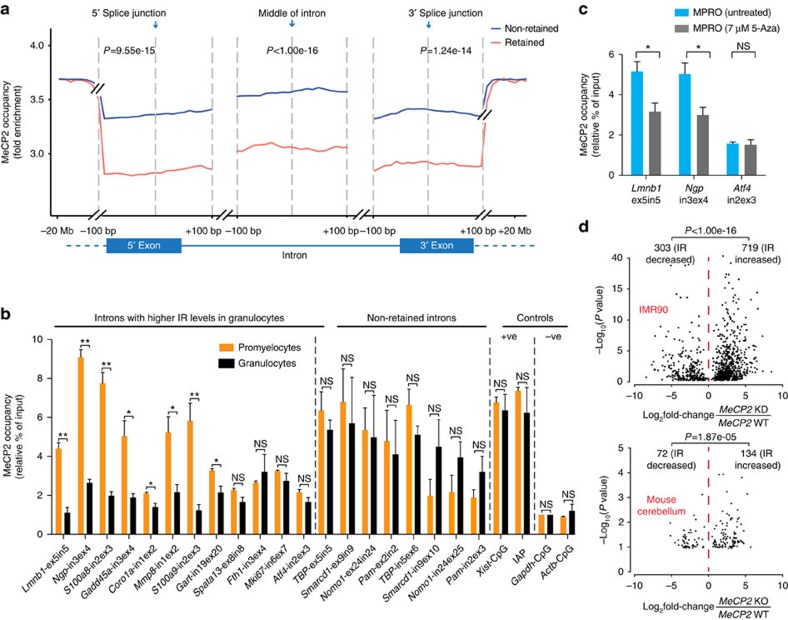
IR is associated with reduced MeCP2 binding near splice junctions. (**a**) Average MeCP2 occupancy normalized against input as measured by ChIP-seq in promyelocytes and granulocytes combined. MeCP2 occupancy is displayed for regions spanning ±100 bp from the 5′ and 3′ splice junctions, and the middle of introns, in the DNA encoding non-retained (blue) and retained introns (red). Data were extended to −20 Mb and +20 Mb from the 5′ and 3′ splice junction respectively. *t*-tests were used to determine significance at *P*<0.05. (**b**) Occupancy of MeCP2 near splice junctions of the DNA encoding introns with increased IR in granulocytes and controls measured by ChIP-qPCR. (**c**) ChIP-qPCR-measured occupancy of MeCP2 near splice junctions of the DNA encoding differentially retained introns of *Lmnb1* and *Ngp* pre- and post- 5-Aza-2′deoxycytidine treatment (5-Aza) in MPRO cells. *Atf4* intron 2 is included as a negative control. (**d**) Association between IR ratio fold changes and their significance following *Mecp2* knockdown in IMR90 cells or *Mecp2*- knockout in primary mouse cerebellum. Binomial test was used to determine the significance of bias between increased and decreased IR at *P*<0.05. For each ChIP-qPCR experiment, a two-tailed Student's *t*-test was used to determine significance, denoted by *P<0.05, **P<0.001 and ns, not significant. Bars display mean±s.e.m. Independent ChIP-qPCR experiments were performed three to five times in triplicate.

Reduced DNA methylation levels near splice junctions can reduce the binding of MeCP2 (ref. 33), which has previously been shown to regulate exon inclusion^12^. To determine whether altered MeCP2 binding is involved in the regulation of IR, we performed genome-wide MeCP2 chromatin immunoprecipitation sequencing (ChIP-seq) in mouse promyelocytes and granulocytes. Consistent with IR-associated reductions in DNA methylation levels (Fig. 1a), a significantly lower density of MeCP2 binding was observed near 5′ and 3′ splice junctions, and in the middle of retained compared to non-retained introns (promyelocytes and granulocytes combined, *P*=9.55e-15, 1.24e-14 and *P*<1.00e-16 respectively, *t*-test, Fig. 3a).

Using chromatin immunoprecipitation-quantitative PCR (ChIP-qPCR), we measured the specific levels of MeCP2 binding near splice junctions flanking twelve introns that are subject to DNA methylation-associated increased IR in granulocytes compared to promyelocytes (Fig. 3b). Significantly reduced levels of MeCP2 binding near splice junctions (*P*<0.05, *t*-test) were observed in 8/12 introns (Fig. 3b) in association with reduced DNA methylation levels at these sites (Fig. 1c,d and Supplementary Fig. 3). No difference in MeCP2 binding was found near splice junctions of four randomly selected non-retained introns, or in the positive and negative controls for MeCP2-ChIP-qPCR (Fig. 3b). In 5-Aza-treated MPRO cells, reduced MeCP2 binding near splice junctions flanking *Lmnb1* intron 5 and *Ngp* intron 3 was also observed (Fig. 3c); concomitant with reduced DNA methylation levels and increased IR levels (Fig. 2c,d). MeCP2 occupancy at the 3′ splice junction of *Atf4* intron 2 did not change (Fig. 3c); consistent with the unaltered low or absent DNA methylation levels following 5-Aza treatment (Supplementary Fig. 4g). These data support the direct involvement of DNA methylation-dependent MeCP2 binding in regulating IR of these genes.

To determine if MeCP2 directly regulates IR more generally, we compared differential IR between matched wild type and *MeCP2*-knockdown and -knockout cells and tissues by analysing publicly available RNA-seq data. Of the differential IR events, we detected predominantly increased IR in all cell and tissue types examined including: IMR90 (719/1022, 70.35%), mouse cerebellum (134/206, 65.05%), mouse peritoneal macrophage (59/91 64.84%) and mouse visual cortex cells (32/46, 69.57%; Fig. 3d and Supplementary Fig. 5a,b). These results indicate that depletion of MeCP2 significantly enhances IR in diverse cell and tissue types (*P*<0.05 in all cases, binomial test, Fig. 3d, and Supplementary Fig. 5a,b).

### IR is associated with increased RNA Pol II stalling

**Figure 4:**
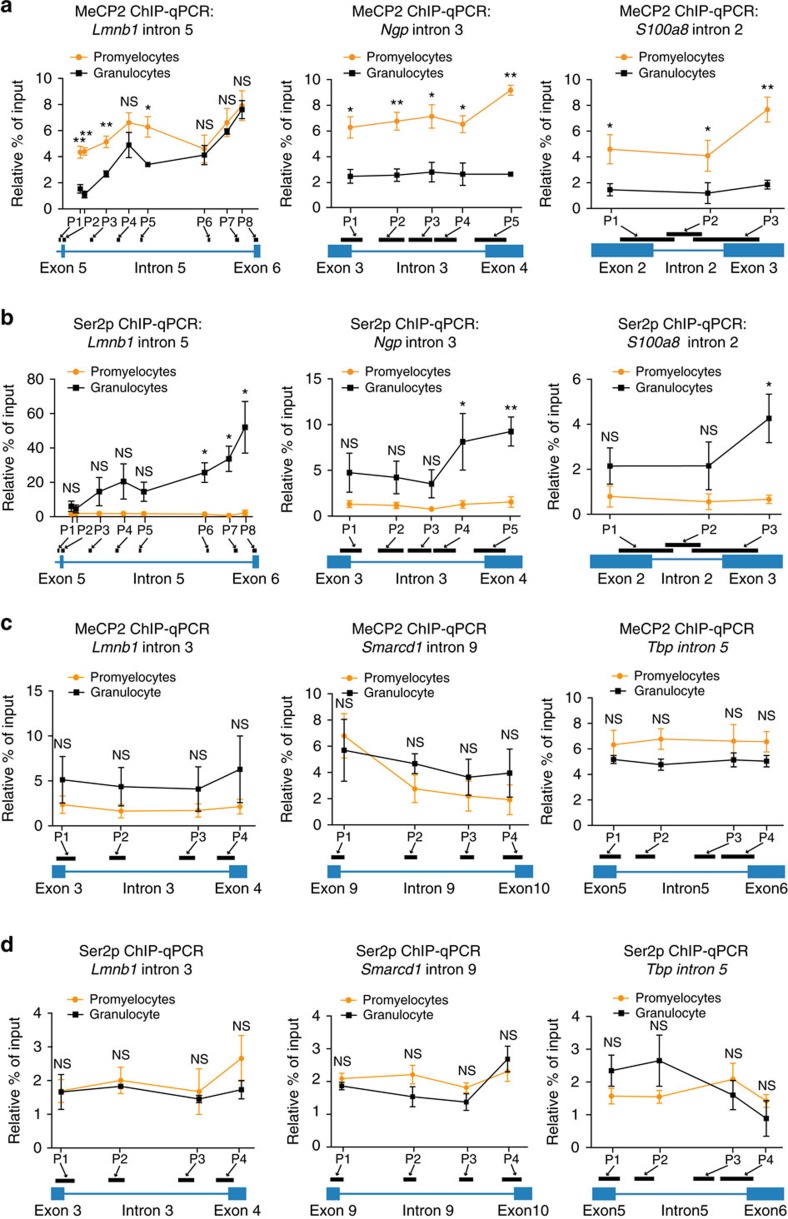
IR is associated with increased RNA Pol II occupancy that anti-correlates with MeCP2 density. (**a**) Occupancies of MeCP2 and (**b**) Pol II Ser2p measured by ChIP-qPCR for retained introns and their flanking exons of *Lmnb1*, *Ngp* and *S100a8* in promyelocytes and granulocytes. (**c**) ChIP-qPCR analyses of MeCP2 and (**d**) Pol II Ser2p occupancies measured for non-retained introns: *Lmnb1* intron 3, *Smarcd1* intron 9, *Tbp* intron 5 and their flanking exons. P1–P8 indicate positions of PCR amplicons within each region. For each ChIP-qPCR experiment, a two-tailed Student's *t*-test was used to determine significance, denoted by *P<0.05, **P<0.001 and NS, not significant. Dots and bars display mean±s.e.m. Independent ChIP-qPCR experiments were performed three to five times in triplicate.

Accumulation of RNA Pol II in alternatively spliced exons can promote their inclusion^13^. This process could be mediated through increased MeCP2 densities within exons, which act as a ‘go slow' signal to promote splice site recognition^12^. Accumulation of the elongating form of RNA Pol II (Pol II Ser2p) near splice junctions has been associated with increased IR levels^9^. We analysed the association between MeCP2 and Pol II Ser2p occupancies within differential intron-retaining genes in promyelocytes and granulocytes using ChIP-qPCR. Consistent with the pattern of reduced DNA methylation (Fig. 1c,d), significantly lower densities of MeCP2 were observed near the 5′ splice junctions of the three introns associated with higher IR levels in granulocytes (*P*<0.05, *t*-test; Fig. 4a). For intron 3 of *Ngp* and intron 2 of *S100a8* in granulocytes, the biggest differences in MeCP2 densities were detected near the 3′ splice junctions (*P*<0.05, *t*-test; Fig. 4a). Pol II Ser2p densities were elevated in introns subject to higher IR levels in granulocytes (Fig. 4b). Furthermore, Pol II Ser2p accumulated at the 3′ splice junctions of all three retained introns (*P*<0.05, *t*-test; Fig. 4b), consistent with a previous analysis of published data^9^. As a control, we noted that MeCP2 and Pol II Ser2p densities were not significantly different between promyelocytes and granulocytes within constitutively spliced intron 3 of *Lmnb1*, intron 9 of *Smarcd1* and intron 5 of *Tbp*, and their flanking exons (Fig. 4c,d). Collectively, our data indicate that lower DNA methylation levels and consequent reduced MeCP2 binding to the DNA that encodes retained introns is associated with increased RNA Pol II stalling within these introns, predominantly near the 3′ splice junctions.

### IR is associated with reduced splicing factor recruitment

**Figure 5:**
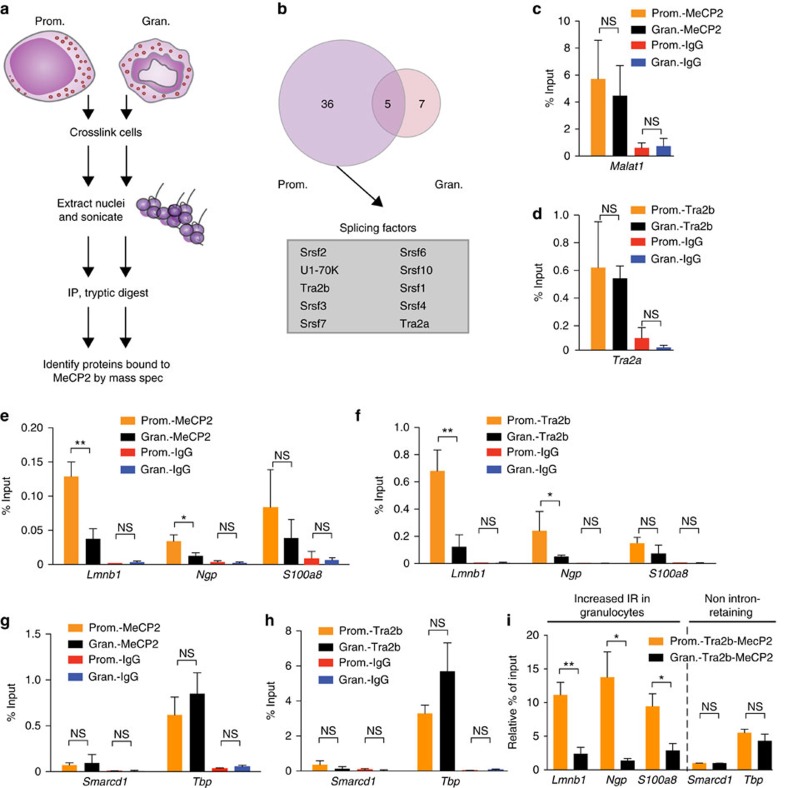
Reduced MeCP2-mediated recruitment of the splicing factor Tra2b promotes IR. (**a**) Schematic of the RIME assay to identify MeCP2 binding partners in normal promyelocytes (Prom.) and granulocytes (Gran.). (**b**) Venn diagram showing proteins that bind to MeCP2 in promyelocytes and/or granulocytes. MeCP2 bound splicing factors enriched in promyelocytes are listed. (**c**) Occupancies of MeCP2 and (**d**) Tra2b in the coding region of *Malat1* and *Tra2a* in promyelocytes and granulocytes measured by RNA-IP followed by qRT-PCR. (**e**) Occupancies of MeCP2 and (**f**) Tra2b near splice junctions of *Lmnb1* intron 5, *Ngp* intron 3 and *S100a8* intron 2 in normal promyelocytes compared to granulocytes by RNA-IP. (**g**) Occupancies of MeCP2 and (**h**) Tra2b near splice junctions of constitutively spliced *Smarcd1* intron 9 and *TBP* intron 5 in normal promyelocytes compared to granulocytes by RNA-IP. (**i**) Occupancies of MeCP2 and Tra2b near splice junctions of *Lmnb1* intron 5, *Ngp* intron 3 and *S100a8* intron 2, *Smarcd1* intron 9 and *TBP* intron 5 in normal promyelocytes compared to granulocytes by RNA-IP-reIP. Levels of occupancies are shown relative to background inferred by sequential Tra2b-IgG RNA-IP controls. A two-tailed *t*-test was used to determine significance, denoted by *P<0.05, **P<0.001 and NS, not significant. Dots and bars display mean±s.e.m. Independent qRT-PCR experiments were performed three to five times in triplicate.

Our results (Fig. 4a,b) indicate an anti-correlation between MeCP2 and Pol II Ser2p densities within introns and near their flanking splice junctions. Thus, the lower MeCP2 levels in granulocytes could not act as a ‘go slow' signal to promote Pol II Ser2p stalling for efficient splice signal recognition. Stalling of Pol II Ser2p has been reported as a consequence of reduced splicing factor recruitment^15^,^16^. Since MeCP2 has previously been shown to bind or recruit splicing factors to pre-mRNAs^34^,^35^,^36^, we hypothesize that Pol II Ser2p stalling in retained introns is consequent to reduced MeCP2-mediated recruitment of splicing factors. To determine splicing factors that associate with MeCP2 in mouse promyelocytes and granulocytes, we performed Rapid Immunoprecipitation Mass Spectrometry of Endogenous Proteins (RIME; Fig. 5a).

RIME identified 39 proteins that co-immunoprecipitated with MeCP2 in promyelocytes and or granulocytes, of which 14 (36%) have previously been reported as MeCP2-binding partners (Supplementary Table 2). Histone and ribosomal RNA proteins are amongst those associated with MeCP2 in these cell types, consistent with previous reports^35^,^37^. Five of these proteins were detected in both cell types (Fig. 5b and Supplementary Table 2). Seven splicing factors (five from the Sr family proteins^38^) co-immunoprecipitated with MeCP2 in promyelocytes but not granulocytes (Fig. 5b and Supplementary Table 2), indicating that one or more of these splicing factors may be associated with MeCP2-mediated splicing events specific to promyelocytes where IR is low. Gene ontology analysis also demonstrated a statistically significant enrichment of splicing-associated functions for proteins that were bound to MeCP2 in promyelocytes but not granulocytes (Supplementary Fig. 5c,d). Of these proteins, Tra2b has recently been shown to be important for efficient AS in human and xenopus cells^39^,^40^, with IR being the dominant mode of splicing affected when Tra2b is knocked down^39^.

We further examined whether reduced MeCP2-mediated Tra2b recruitment could account for reduced splicing leading to IR. Another consequence of this model would be that Tra2b is recruited by MeCP2 to promote splicing in promyelocytes. There is no substantial difference in the MeCP2 and Tra2b protein levels in promyelocytes and granulocytes (Supplementary Fig. 5e,f). RNA immunoprecipitation (RNA-IP) followed by qPCR with reverse transcription (RT-qPCR) showed that binding of MeCP2 and Tra2b to their known interacting RNA, *Malat1* and *Tra2a*, was not significantly different in promyelocytes and granulocytes (Fig. 5c,d). In contrast, there was a significantly reduced association of MeCP2 with *Lmnb1* and *Ngp* transcripts, both of which showed higher IR levels in granulocytes (*P*<0.05, *t*-test; Fig. 5e). A non-significant trend of reduced MeCP2 binding was also observed at *S100a8* in granulocytes (Fig. 5e). Importantly, the pattern of Tra2b enrichment copied that of MeCP2 (Fig. 5f), indicating that these two proteins function together to promote splicing. The levels of association between MeCP2 and Tra2b with constitutively spliced *Smarcd1* and *Tbp* transcripts were not significantly different in promyelocytes and granulocytes (Fig. 5g,h).

We further investigated whether Tra2b and MeCP2 act together in a complex that regulates splicing by performing sequential RNA-IP with antibodies against Tra2b and MeCP2 (RNA-IP-reIP). Our RNA-IP-reIP assays consistently detected de-enrichment of concomitant Tra2b and MeCP2 binding to *Lmnb1*, *Ngp* and *S100a8* transcripts that increased in IR levels in granulocytes compared to promyelocytes (*P*<0.05, *t*-test; Fig. 5i). The levels of concomitant Tra2b and MeCP2 binding to non intron-retaining *Smarcd1* and *Tbp* transcripts were not significantly different (Fig. 5i). Thus, when MeCP2 and Tra2b reduce their interaction with mRNA there is a consequent increase in IR.

**Figure 6:**
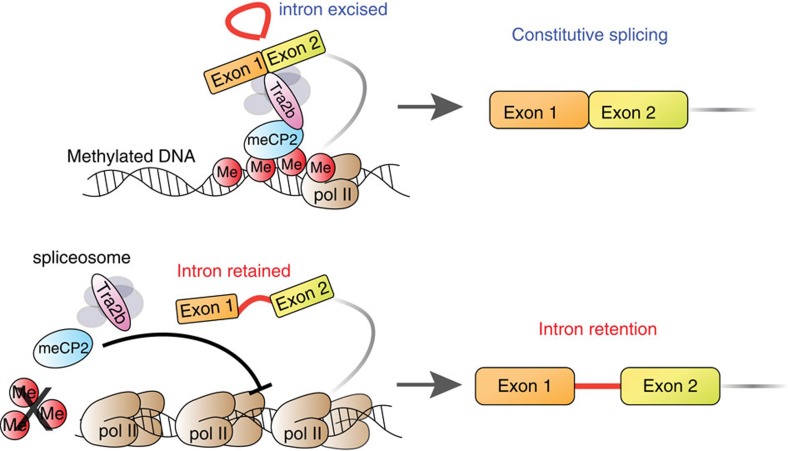
Proposed model of IR regulation. IR occurs via less efficient splicing factor (e.g. Tra2b) recruitment consequent to reduced DNA-methylation-mediated MeCP2 binding.

Our data support a model where MeCP2 regulates splicing via recruitment of splicing factors, such as Tra2b (Fig. 6). IR may occur in cases where lack of MeCP2 binding to the DNA and RNA results in less efficient recruitment of the Tra2b and potentially other splicing factors (Fig. 6).

## Discussion

Until recently, IR has been the least understood mode of splicing in terms of its role in normal physiology and disease. As a result of recent work by our group and others, it is now established that IR is a conserved mechanism that regulates cell differentiation and plasticity in normal tissues and is implicated in diverse human cancers^1^,^2^,^8^,^9^,^41^,^42^,^43^. For example, IR underlies normal differentiation and physiological cellular functions in granulopoiesis, erythropoiesis, megakaryopoiesis and lymphopoiesis^2^,^7^,^8^,^44^. It is also a mechanism of tumour suppressor gene inactivation in diverse cancers^43^. In this work we sought to understand the molecular mechanisms underlying IR as a foundation to establish why specific introns are retained whereas others are not.

Since DNA methylation is known to regulate other forms of splicing, we explored its role in controlling IR. Two models of splicing regulation via DNA methylation have been proposed: ‘kinetic' and ‘recruitment'. The former involves ‘go slow' signal proteins reflecting DNA methylation changes, to reduce the rate of RNA Pol II elongation and promote recognition of splice sites^12^,^13^. The ‘recruitment model' applies to cases where DNA methylation directly recruits splicing factors to promote splicing^18^. Our results demonstrate a previously unrecognized link between the ‘kinetic' and ‘recruitment' models of splicing regulation, revealed by examining protein complexes associated with DNA methylation. We have established that reduced DNA methylation decreases the binding of MeCP2 to the DNA and RNA, which in turn reduces splicing and consequently increases IR (Figs 3 and 4). In support of this model we showed that splicing factors, such as Tra2b, are less efficiently recruited to RNA, and are associated with RNA Pol II stalling near the splice junctions flanking retained introns and within retained introns themselves (Fig. 4). Subsequently, splicing repressors may be recruited following RNA Pol II stalling to stabilize IR (ref. 9).

Our data indicate that DNA methylation changes are associated with approximately one in every six differential IR events in myeloid cells. This observation reveals the complexity of splicing and IR regulation, which may involve many different epigenetic factors and associated proteins. Indeed, H3.3K36me3 mediated recruitment of BS69 is known to promote IR by antagonizing the activity of the spliceosome via its interaction with snRNPs, including EFTUD2 (ref. 10). While BS69 has also been shown to repress RNA Pol II elongation^45^, this work was performed in U20S cells rather than HeLa cells where the IR-promoting role of BS69 was reported. Thus, the possibility that BS69 plays a dual role in linking the ‘kinetic' and ‘recruitment' models of splicing remains to be determined. BS69 also binds to several core splicing factors including PRPF8, SRSF1 and SRSF4 (ref. 10). BS69 may act as a physical barrier that prevents splicing factors from accessing nascent RNA while promoting the recruitment of splicing repressors via inhibition of transcription elongation. Alternatively, repression of transcription elongation may result from less efficient splicing factor recruitment, as we proposed in our model of IR regulation based on DNA methylation and MeCP2 (Fig. 6).

It is important to recognize that AS can also be modulated by the ratio of DNA methylation to hydroxymethylation^46^. While we have utilized bisulfite sequencing, which cannot discriminate between DNA methylation and DNA hydroxymethylation, the levels of DNA hydroxymethylation in granulocytes are low^47^. Thus, the majority of unconverted cytosines we detected in granulocytes following bisulfite sequencing are most likely methylated cytosines. Whether DNA hydroxymethylation also regulates IR is unknown. This question will need to be explored in cells or tissue with high levels of DNA hydroxymethylation, and using sequencing methods that specifically detect this type of DNA modification^48^.

Although intragenic DNA demethylation has been associated with reduced gene expression in cancers^49^, it remains to be shown whether this occurs via aberrant IR-triggered NMD. The DNA methylation inhibitor, 5-Aza, is FDA-approved for the treatment of myelodysplastic syndromes and is in clinical trials for diverse cancers^50^,^51^. The extensive association between reduced DNA methylation and increased IR that we have shown in diverse tissue types indicates that global demethylation induced by 5-Aza may exert effects, which promote aberrant IR.

In conclusion, we have discovered a causative role of DNA methylation changes in the regulation of IR via MeCP2 binding and splicing factor recruitment. Our findings reveal another mechanism that controls splicing and IR in diverse tissue types. Further understanding of this mechanism in the development of cancers, and its impact in patients receiving DNA methylation inhibitors may contribute to improved therapeutic strategies.

*Note added in proof:* At the proof stage of this manuscript, our software article detailing the IRFinder algorithm, Middleton *et al*.^52^, was published.

## Methods

### Primary cells and cell lines

Eight–10-week-old C57BL6/J mice were obtained from the Animal Resource Centre (Perth, Australia). All mouse experiments were performed in a specific pathogen-free facility with approval from the Royal Prince Alfred Hospital Animal Ethics Committee (Protocol no. 2013/045). The FACS strategies for promyelocytes and granulocytes are shown in Supplementary Fig. 1a as previously published^2^,^53^. Briefly, harvested bone marrow cells were incubated with lineage specific antibodies conjugated to biotin: B220 (BioLegend 103204, 1:200), CD19 (BioLegend 115504, 1:200), CD3 (BioLegend 100304, 1:200), Sca–1(BioLegend 108104, 1:200), together with anti–Gr–1–fluorescein isothiocyanate (FITC; Biolegend 108406, 1:200), anti–cKit–phycoerythrin (PE; Becton Dickinson 553355, 1:200), anti–CD34–Alexafluor 660 (eBioscience 50-0341-82, 1:100) and anti–CD16/32–PerCP/Cy5.5 (Becton Dickinson 560540, 1:400) antibodies. Cells were washed twice in phosphate-buffered saline (PBS) with 2% (volume per volume) foetal bovine serum (HyClone, GE Healthcare Life Sciences) and incubated with streptavidin–APC–Cy7 (BioLegend 405208, 1:1,000). Following two additional washes in PBS with 2% (volume per volume) foetal bovine serum, the cells were sorted into 15 ml collection tubes on a BD Influx Cell Sorter (BD Biosciences). Morphological confirmation of promyelocytes and granulocytes was performed following May-Grünwald Giemsa staining of cells spun onto poly-L-lysine slides (Supplementary Fig. 1b). MPRO cells were maintained in DMEM media supplemented with 5% foetal bovine serum (Hyclone, GE Healthcare Life Sciences), 10% BHK-HM5 conditioned medium made in-house by collecting and filtering the DMEM media in which BHK/HM5 cells were cultured for 96 h to produce granulocyte macrophage colony-stimulating factor, 5 U ml^−1^ penicillin, 5 μg per ml streptomycin sulfate and 2 mM L-glutamine.

### DNA methylation analysis

Genomic DNA extracted from cells was bisulfite-converted using the EZ DNA Methylation-Gold Kit (Zymo Research, Orange, CA, USA), according to the manufacturers' instructions. For WGBS analyses, libraries were prepared from bisulfite-converted samples and sequenced on an Illumina HiSeq2000 platform at Macrogen Inc. (Korea). For gene-specific clonal bisulfite sequencing, bisulfite-converted DNA was amplified using primers that equally amplify methylated and unmethylated template. Primer sequences are provided in Supplementary Table 3. PCR amplicons were cloned into pGEM-T Easy vectors (Promega, Madison WI USA) and sequenced using Sanger Sequencing.

### Inhibition of DNA methylation

Inhibition of DNA methylation in MPRO cells was performed using 3 μM or 7 μM 5-Aza-2′ deoxycytidine for 120 h with drugs replenished every 24 h. Dead cells were excluded by FACS following 4′,6-diamidino-2-phenylindole staining.

### RNA isolation and qRT-PCR analyses

Total RNA extraction was performed using Trizol (Invitrogen). RNA 6000 Nano Chips (Agilent Technologies) were used to confirm RNA quality. For qRT-PCR, total RNA was treated with DNAse I, amplification grade (Invitrogen). cDNA was generated using SuperScript III First-Strand Synthesis System (Invitrogen), according to the manufacturers' instructions. qRT-PCR was performed using the CFX96 Real Time PCR Machine (Bio-Rad). Reactions were performed in 20 μl volumes containing 1 × IQ SyberGreen supermix and 0.3 μM of the respective forward and reverse primers. Cycling conditions were: 95 °C for 6 min followed by 95 °C for 30 s, primer-specific annealing temperature for 30 s, and extension at 72 °C for 30 s. Primer sequences are shown in Supplementary Table 3.

### Inhibition of NMD using caffeine coupled with actinomycin D

NMD inhibition was performed on MPRO cells after 5-Aza-2′ deoxycytidine treatment as previously published^2^. Briefly, cells in culture media were treated with caffeine (15 mM) for 4 h before they were washed twice with PBS. Caffeine and actinomycin D (2 μg ml^−1^) were then added, and an actinomycin D-only control was used to block transcription of genes. Following an additional 4 h of incubation, total RNA was collected for qRT-PCR.

### Western blot

Total protein lysates were loaded onto precast SDS-PAGE gels (Invitrogen) and subjected to electrophoresis before being transferred onto polyvinylidene difluoride (PVDF) membranes. Membranes were blocked with 5% (weight per volume) skim milk for 1 h at room temperature and incubated overnight with a rabbit anti-MeCP2 (5 μg, Abcam ab2828; Supplementary Fig. 5e, a rabbit anti-Tra2b (10 μg, Abcam ab31353; Supplementary Fig. 5f), or a mouse anti-Gapdh (1:1,000, Abcam ab8245; Supplementary Fig. 5e,f). Following washes, membranes were incubated with HRP-conjugated secondary anti-rabbit or anti-mouse antibody (1:5,000; Chemicon AP182P or AP192P), and exposed using enhanced chemiluminescence reagents (Pierce) on a Kodak Imager (Kodak). Bands were quantified using ImageJ. All uncropped blots are displayed in Supplementary Fig. 6.

### Chromatin immunoprecipitation

ChIP was performed with either the Magna ChIP A/G kit (Millipore 17-10085) or Magna ChIP HiSens kit (Millipore 17-10460) following the manufacturers' instructions, with minor modifications. Briefly, 5 × 10e^6^ FACS-enriched promyelocytes or granulocytes were cross-linked with 0.5% formaldehyde for 10 min at room temperature, and quenched with 1 × glycine for another 5 min. Cells were washed twice with ice-cold PBS, lysed and sonicated using Bioruptor Plus (Diagenode, UCD-300) with 15 cycles of 30 second ON and 30 s OFF at high power setting. Sonicated samples were centrifuged for 10 min at 10,000*g*. Supernatant was incubated overnight at 4 °C with the antibody of interest mixed with protein A/G magnetic beads. Following washes, crosslinking was reversed at 62 °C for 2 h and DNA was purified using the Agencourt AMPure XP beads (Beckman Coulter, A63881). Antibodies used were rabbit anti-MeCP2 (5 μg, Abcam ab2828), rat anti-Pol II Ser2p (7.5 μg, Active Motif 61084), rabbit IgG control (5 μg Abcam ab46540) and normal rat IgG (7.5 μg, Santa Cruz sc-3036). ChIP DNA was analysed by qPCR with primers listed in Supplementary Table 3. ChIP-qPCR results were reported as percentage of input relative to negative controls comprising a CpG-rich promoter region of Gapdh and an intergenic region on mouse chromosome 6 for MeCP2 and Pol II Ser2p respectively.

For ChIP-seq, sample concentration and size distribution of immunoprecipitated DNA were measured with Qubit dsDNA HS Assay Kits (Invitrogen) and High Sensitivity DNA Analysis Kit (Agilent Technologies), respectively. Approximately 20 ng of the input and ChIP DNA samples were sent to Macrogen Inc. (Korea) for ChIP-seq library preparation and sequencing on an Illumina Hiseq2000 platform.

### Rapid immunoprecipitation and mass spectrometry

Rapid immunoprecipitation and MS was performed as previously reported^54^, except that the chromatin immunoprecipitation step was performed using the Magna Chip A/G kit (Millipore 17-10085). Briefly, cells were sonicated and incubated overnight with antibody of interest mixed with protein A/G magnetic beads as described in the chromatin immunoprecipitation Method section. Following washing of antibody–protein-beads complex with buffers provided in the kit, trypsin digestion was perform to release the bound peptides. MS was performed on a QExactive Plus (Thermo Fisher). The data were searched within the Mouse SwissProt database using the Mascot search engine (version 2.4.0) with tryptic specificity allowing up to two missed cleavages, peptide mass tolerance of 10 p.p.m. and 0.8 Da for MS/MS ion mass tolerance. Variable modifications were oxidized methionine, acetylated asparagine and carbamidomethylated cysteine. IgG controls were used to exclude non-specific interactions. Only proteins present in three out of three biological replicates and not in any of the IgG controls were reported.

### RNA immunoprecipitation

RNA-IP was performed using the Magna Nuclear RNA-IP (cross-linked) kit (17-10520) from Millipore according to the manufacturer's protocol. Antibodies used were rabbit anti-MeCP2 (10 μg, Abcam ab2828) and rabbit anti-Tra2b (10 μg, Abcam ab31353). Approximately 1 million 0.5% formaldehyde-fixed cells were used for RNA-IP. Immunoprecipitated RNA was purified using Trizol LS Reagent (Life Technologies, 10296-010) and subjected to DNase I treatment. iTaq universal SYBR Green one-step kit (Bio-Rad) was used to quantify the amount of immunoprecipitated RNA. Primers used were listed in Supplementary Table 3.

### RNA immunoprecipitation re immunoprecipitation (RNA-IP-reIP)

RNA-IP-reIP was performed using the same kit used for RNA-IP: Magna Nuclear RNA-IP (Cross-linked) kit (17-10520) from Millipore. Approximately 2 million 0.5% formaldehyde-fixed cells were used for each RNA-IP-reIP. Sequential RNA-IP was performed with an additional purification step between the two immunoprecipitation steps. Briefly, immunoprecipitation was performed on fixed and sonicated chromatin lysate using the rabbit anti-Tra2b antibody (20 μg, Abcam ab31353) together with A/G magnetic beads at 4 °C for 1 h. Following washes according to the manufacturer's instruction, the bead bound RNA-Protein complex were released with the elution buffer supplemented with DTT, RNase inhibitor and protease inhibitor for 15 min at 37 °C. SDS was removed from the eluent with HiPPR Detergent Removal spin column (Thermo Fisher, PIE88305) before the subsequent RNA-IP.

The eluent from the first RNA-IP was then used for a second RNA-IP at 4 °C for 1 h with either the Rabbit anti-MeCP2 (10 μg, Abcam ab2828) or the normal rabbit IgG (10 μg, Santa CruzSC-2027) as control. Following washes as recommended by the manufacturer, the immunoprecipitated RNA was purified using the Trizol LS Reagent (Life Technologies, 10296-010) and DNase I treatment was performed. iTaq universal SYBR Green one-step kit (Bio-Rad) was used to quantify the amount of immunoprecipitated RNA relative to the sequential Tra2b-IgG RNA-IP control. The workflow for RNA-IP-reIP is shown in Supplementary Fig. 7. Primers used were listed in Supplementary Table 3.

### Bioinformatics analyses

Besides the WGBS and RNA-seq reads generated from promyelocytes and granulocytes, we also analysed WGBS, RNA-seq and MeCP2 ChIP-seq data from the GEO (Gene expression Omnibus) database (Supplementary Table 1). All RNA-seq datasets have been generated from poly-A enriched libraries as published elsewhere^2^,^12^,^55^,^56^,^57^. To assess genome-wide DNA methylation data, WGBS data was aligned to mouse genome GRCm38/mm10 and human genome GRCh37/hg19, respectively, followed by the calculation of methylation level (beta value) at each CpG site using Bismark v0.9.0 (ref. 50) with default parameters. Only those CpG sites covered by more than five reads were considered as valid for the downstream analysis. A sliding window of 10 bp was then applied to scan the ±100 bp region of a splice junction or from the middle of an intron. Within each window, average methylation level was calculated as the sum of beta values divided by the number of CpGs. For gene expression analysis, TopHat v2.2.2 with default parameters was applied to map RNA-seq reads against Ensembl human (GRCh37.75) and mouse (GRCm38.73) transcriptomes^58^. Cufflinks v2.1.1 was then applied to measure the FPKM of each gene^58^.

To assess the MeCP2 binding density, ChIP-seq reads were mapped to human (hg19) or mouse (mm10) genomes using Bowtie2 v2.2.3 default settings. MAC2 (ref. 59) was applied to call MeCP2 peaks from mapped ChIP-seq reads, with an extension size of 250nt and a *q*-value (FDR) cut-off of 0.05. To assess the MeCP2 binding density, ChIP-seq reads were mapped to human (hg19) or mouse (mm10) genomes using Bowtie2 v2.2.3 with default settings. MACS2 (ref. 54) was applied to call MeCP2 peaks from mapped ChIP-seq reads, with an extension size of 250nt and a *q*-value (FDR) cut-off of 0.05. MeCP2 density at each peak was reported as fold enrichment, which is the number of position-adjusted reads in MeCP2-ChIP sample/input. A sliding window of 10 bp was then applied to scan the ±100 bp region of a splice junction or from the middle of an intron. Within each window, average MeCP2 signal was calculated as the sum of fold enrichment divided by the number of peaks.

### Intron retention discovery

The steps of detecting IR in an individual sample and alternate IR between samples are described below:

A STAR^60^ genome index is built with the human or mouse genome fasta file (GRCh37/GRCm38) and annotation gtf file (Ensembl 75 for human, 78 for mouse). Features that overlap with introns such as microRNAs or snoRNAs are excluded as these may confound the accurate measurement of true intron levels. To accurately measure the expression levels of introns, intronic regions of poor mappability are determined by mapping synthetic reads to the genome and are excluded from further analysis. Synthetic reads are taken from the human or mouse genome reference, which are 70 bp long and stepped at 10 bp across the entire genome. Every second read is reverse complemented. A single base error is generated in the centre of each read. Those synthetic reads uniquely mapping to the correct location are tallied. Any area without at least 5 out of a possible 7 unique reads is considered poorly mappable. Poorly mappable regions are excluded from the measurable intron area regardless of strand or direction.

RNA-seq reads were aligned to the reference genome by STAR using default scoring parameters but excluding multi-mapping reads from the output. The default scoring parameters suit the detection of IR as they favour neither mapping from exon to exon across splice junctions nor mapping from exons into introns. IR is then determined by measurement of both the splicing level and intronic abundance. The key calculated metric is the IR ratio. Abundance of normal splicing is measured by a count of read fragments spliced across the intron. Reads that start in the 5′ exon but end in another exon and reads that start in another exon but end in the 3′ exon are also counted, the larger value being used. Intronic abundance is measured as a trimmed mean of fragment coverage depth. The trimmed mean excludes previously excluded regions (that is, regions with poor mappability, miRNAs or ncRNAs) and the highest and lowest 30% of values. This compensates for noise in intronic coverage. The IR ratio is calculated simply as follows:


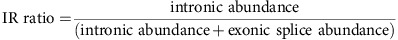


To compare the change of IR of a certain intron between a pair of samples or groups, the intronic abundance and exonic splice abundance of this intron is evaluated using a Bayesian statistic adapted for digital counts^61^. Significance of the change in IR ratio is considered using the following cut-off: 1) *P*-value is <0.05; 2) exonic splice abundance for both samples or groups are above 10; 3) IR ratio in one sample or group is above 0.1.

### Gene ontology enrichment analysis

Gene ontology enrichment analysis was performed using DAVID^62^. Gene ontology categories with a *P*-value <0.01 after Benjamini Hochberg correction were considered significant.

### Statistical analysis

Statistical analyses were performed as indicated in the text and/or figure legends. To compare the overall DNA methylation level or MeCP2 fold enrichment across the ±100 bp flanking splice junctions, the whole region (200 bp) was first divided into 20 sub-windows (10 bp each). Within each sub-window, the mean of DNA methylation level or MeCP2 fold enrichment for all IR and non-IR events was calculated respectively and paired together. Paired *t*-test was then applied to assess the significance of overall difference across all the sub-windows.

The significance of the difference between the number of increased and decreased IR events in cells following DNA methylation inhibition or MeCP2 knockdown was determined using the binomial test. Student *t*-tests were used to compare differential gene expression and levels of GC content and CpG density between retained and non-retained introns. Differential enrichment of MeCP2, Pol II Ser2p and Tra2b assessed by qPCR following ChIP and/or RNA-IP was also determined using *t*-tests. *F*-test was used to determine the significance of regression analysis. Analyses were performed using Graph-Pad Prism v.6 (La Jolla, CA, USA). *P*<0.05 was considered significant for all tests.

### Data availability

WGBS and ChIP-seq data that support the findings of this study have been deposited in Gene Expression Omnibus with the accession number GSE85517. All other data that support the findings of this study are available from the corresponding authors upon reasonable request.

## Additional information

**How to cite this article:** Wong, J. J.-L. *et al*. Intron retention is regulated by altered MeCP2-mediated splicing factor recruitment. *Nat. Commun.* **8**, 15134 doi: 10.1038/ncomms15134 (2017).

**Publisher's note:** Springer Nature remains neutral with regard to jurisdictional claims in published maps and institutional affiliations.

## Supplementary Material

Supplementary Information Supplementary Figures and Supplementary Tables
