# Supplementary material for: Intron retention is regulated by altered MeCP2-mediated splicing factor recruitment
Source: Nat Commun. 2017 May 8;8:15134. doi: 10.1038/ncomms15134 (PMC5424149; doi:10.1038/ncomms15134)
Supplement: Supplementary Information — Supplementary Figures and Supplementary Tables [file ncomms15134-s1.pdf]

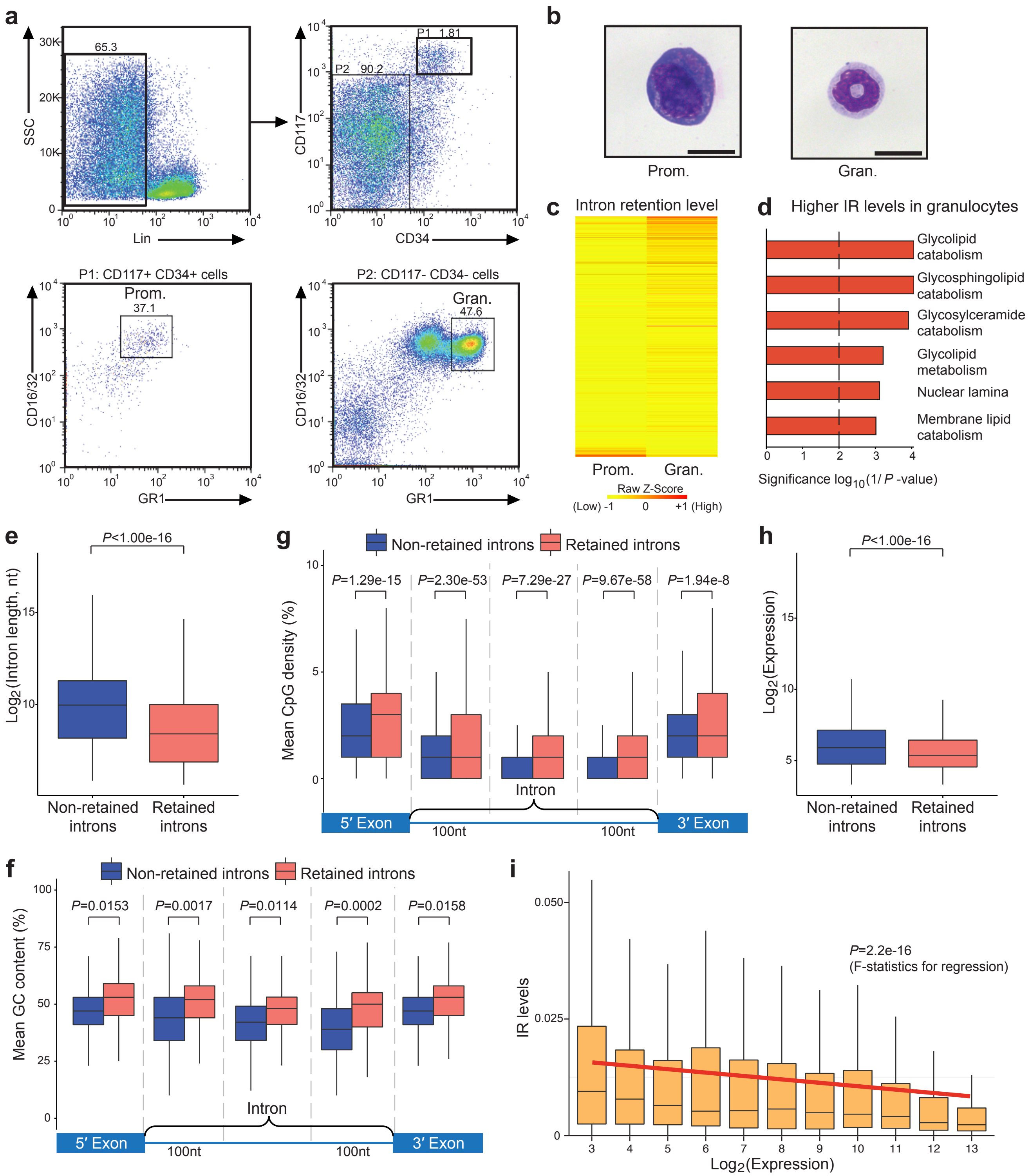

Supplementary Figure 1. Features associated with IR in promyelocytes and granulocytes. (a) Fluorescence-activated cell sorting strategy to isolate Lin-Kit+CD34+CD16/32+Gr-1low promyelocytes (Prom.) and Lin-Kit-CD34-CD16/32+Gr-1high granulocytes (Gran.) from mouse bone marrow. **(b)** Representative morphology of a promyelocyte and a granulocyte examined using a light microscope at 100× magnification following staining with May Grünwald Giemsa. Scale bars indicate 10 µm. **(c)** Heatmaps generated from RNA-seq data showing the expression levels of differentially-retained introns in promyelocytes and granulocytes. **(d)** Gene ontology enrichment of the genes with significantly increased IR levels in granulocytes (P<0.01 with Benjamini-Hochberg correction). **(e)** Box and whisker plots displaying length distribution of non-retained versus retained introns in promyelocytes and granulocytes. **(f)** GC content and **(g)** CpG density for introns and flanking exons of non-retained versus retained introns spanning ±100bp from the 5′ and 3′ splice junctions, and from the middle of introns. **(h)** Expression levels of the exons flanking non-retained and retained introns. **(i)** A series of box and whisker plots displaying the association of IR levels with gene expression in promyelocytes and granulocytes. Genes with a similar expression level were binned into categories shown on the x-axis. F-test was applied to determine the fitness of the regression model (red line) at P<0.05.

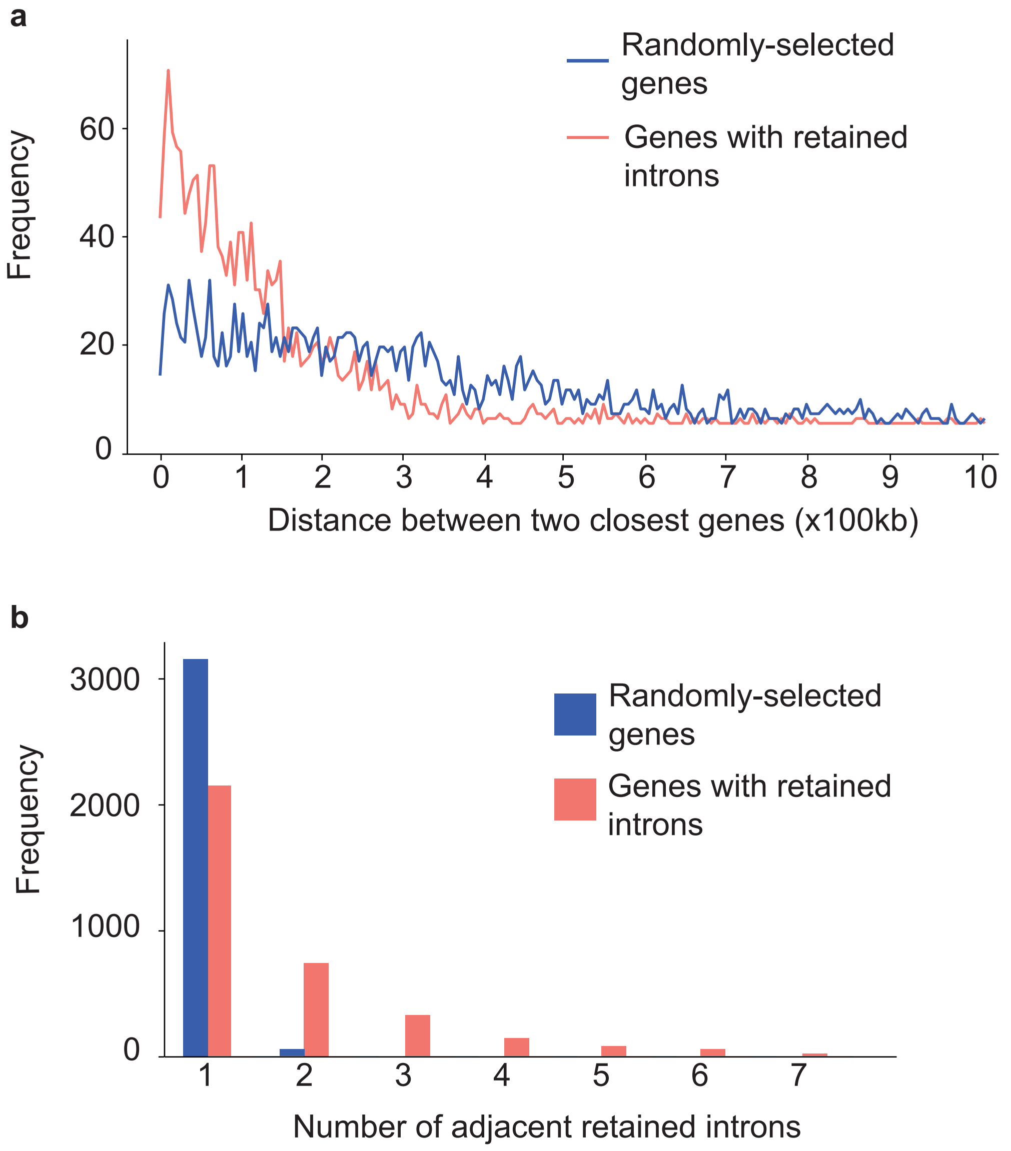

Supplementary Figure 2. Retained introns within genes and genomic positions of genes with retained introns are clustered. (a) Genomic distance between 2 closest genes with retained introns in granulocytes (n=1739) and in a control set where the same number of genes were randomly selected amongst genes expressed in granulocytes (>10 Fragments Per Kilobase of transcript per Million mapped reads, FPKM). **(b)** Frequency of adjacent retained introns, separated only by one or more annotated exon(s) within the same gene in granulocytes compared to a control set where the same number of introns were randomly selected within the same gene.

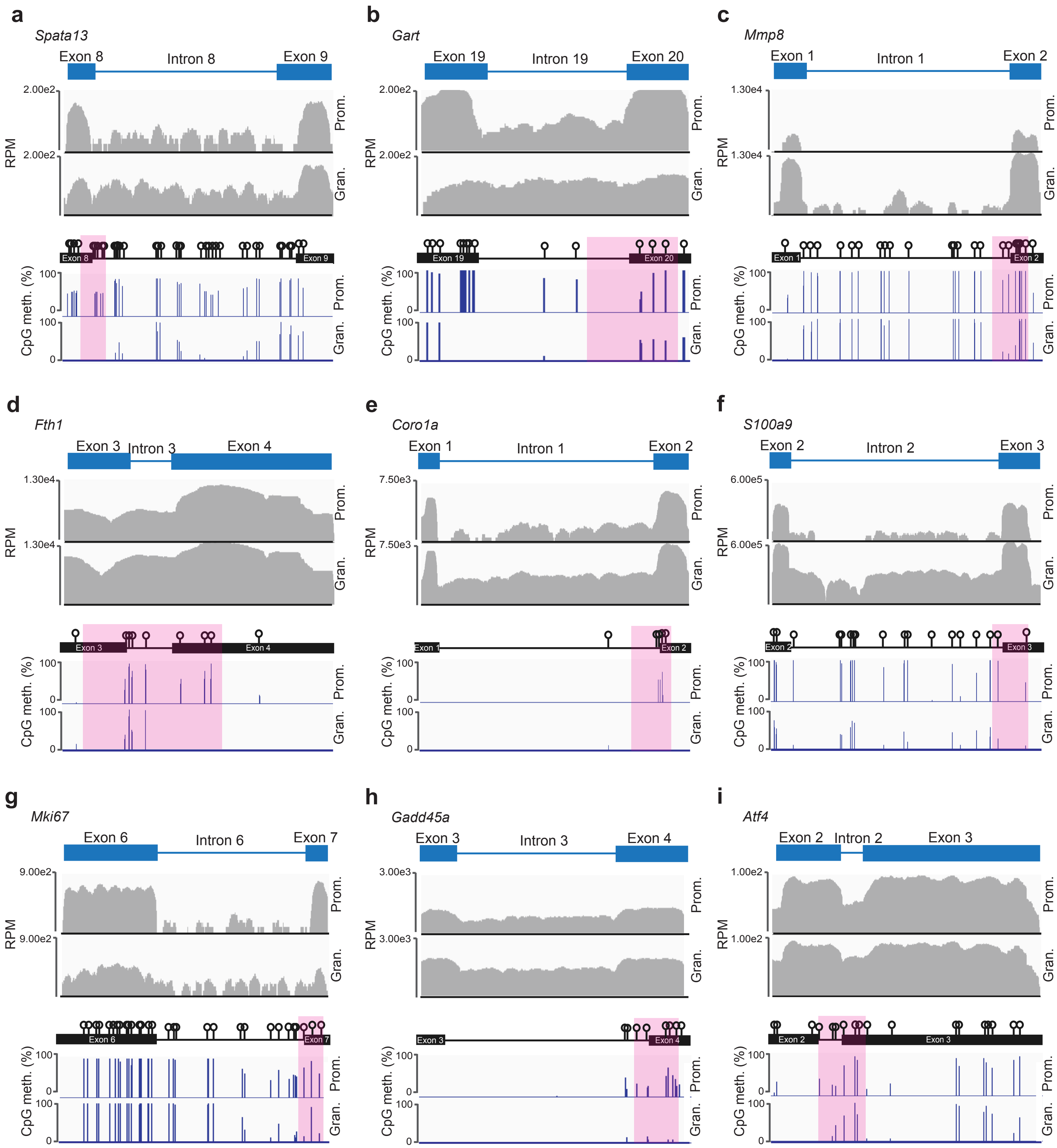

Supplementary Figure 3. Higher levels of IR in granulocytes are associated with lower levels of DNA methylation near 5' and/or 3' splice junctions. RNA-seq reads (RPM values) within introns and flanking exons in promyelocytes (Prom.) and granulocytes (Gran.) shown on the same scale using IGV v2.3 software for murine genes **(a)** *Spata13*, **(b)** *Gart*, **(c)** *Mmp8*, **(d)** *Fth1*, **(e)** *Coro1a*, **(f)** *S100a9*, **(g)** *Mki67*, **(h)** *Gadd45a* and **(i)** *Atf4*. Shown beneath the mRNA-seq data are average methylation levels (0-100%) at individual CpGs (indicated by lollipops on the maps) detected by WGBS within specific introns and flanking exons of these genes. A ±100bp region around splice junctions, with reduced DNA methylation in granulocytes, is shaded in pink.

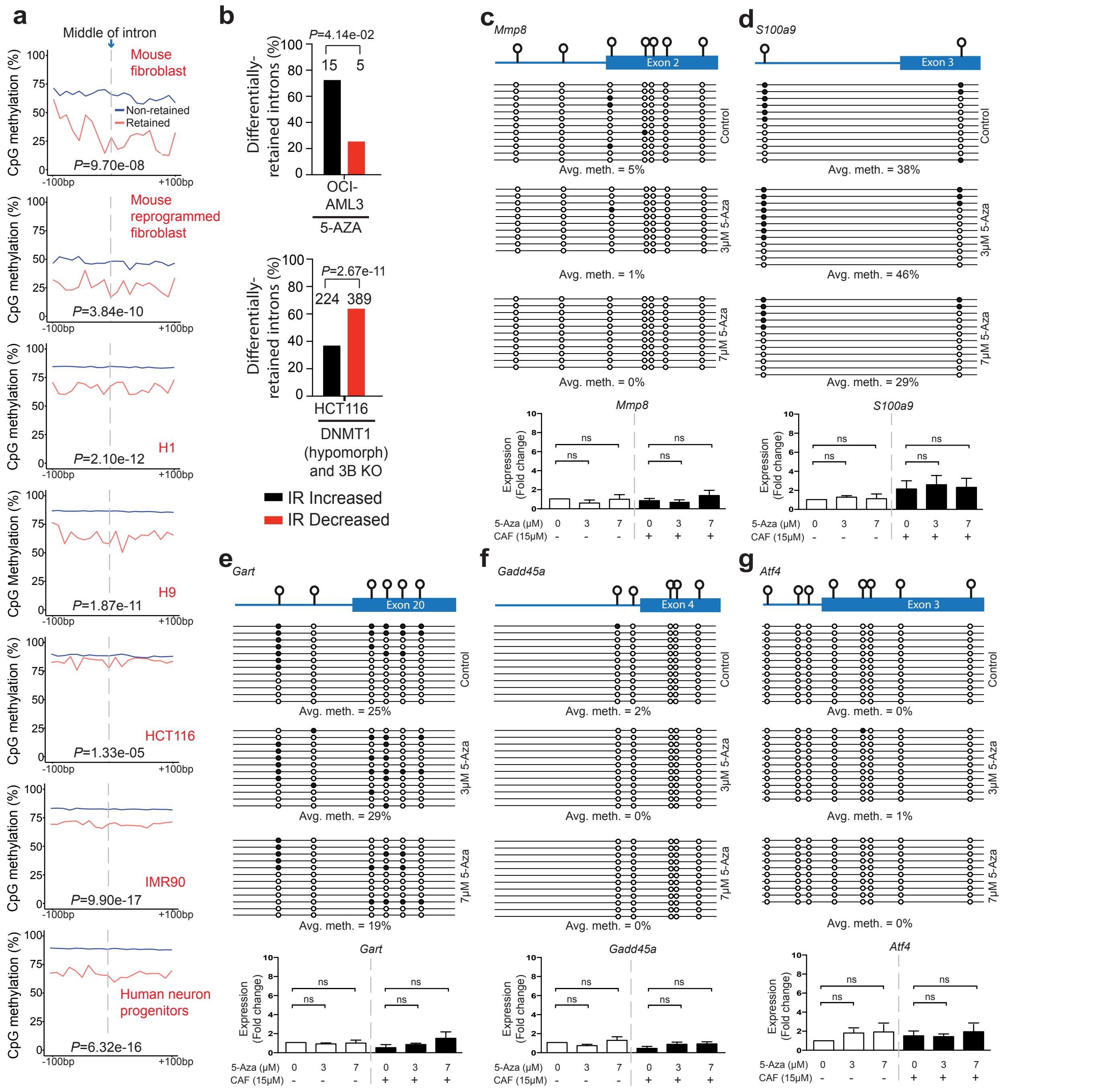

Supplementary Figure 4. Increased IR occurs consequent to reduced DNA methylation levels. (a) Average DNA methylation levels spanning ±100bp from the middle of the intron in the DNA encoding non-retained (blue) and retained (red) introns in primary mouse fibroblasts, mouse reprogrammed fibroblasts, human cell lines (H1, H9, HCT116, IMR90) and human primary neuron progenitors. **(b)** Percentage (%) of introns in OCI-AML cells treated with 5-Aza-2′deoxycitidine (5-Aza) or engineered DNMT3B and hypomorphic DNMT1 double-KO HCT116 cells that increased (black) or decreased in IR (red) compared to control. Number of events with increased or decreased IR is shown above the bars. Binomial test was used to determine significance at $P<0.05$. **(c-g)** Clonal bisulfite sequencing displaying DNA methylation changes near 3′ splice junctions of *Mmp8* intron 1, *S100a9* intron 2, *Gart* intron 19, *Gadd45a* intron 3 and *Atf4* intron 2 in MPRO cells following treatment with 3 and 7 μM 5-Aza. Each row represents a single cloned PCR amplicon aligned to the exon-intron map shown. Each lollipop on the map represents a CpG site. Black and white circles denote methylated and unmethylated CpGs respectively. Lower panel: IR levels determined by qRT-PCR for the same indicated introns in MPRO cells, with and without exposure to 5-Aza at 3 or 7 μM, and in the absence (-) or presence (+) of NMD inhibition via caffeine (CAF) treatment. Two-tailed t-test was used to determine significance at $P<0.05$. Bars display mean ± standard error of the mean. Independent qRT-PCR experiments were performed three times in triplicate (n=3). ns, not significant; Avg. meth., average methylation levels.

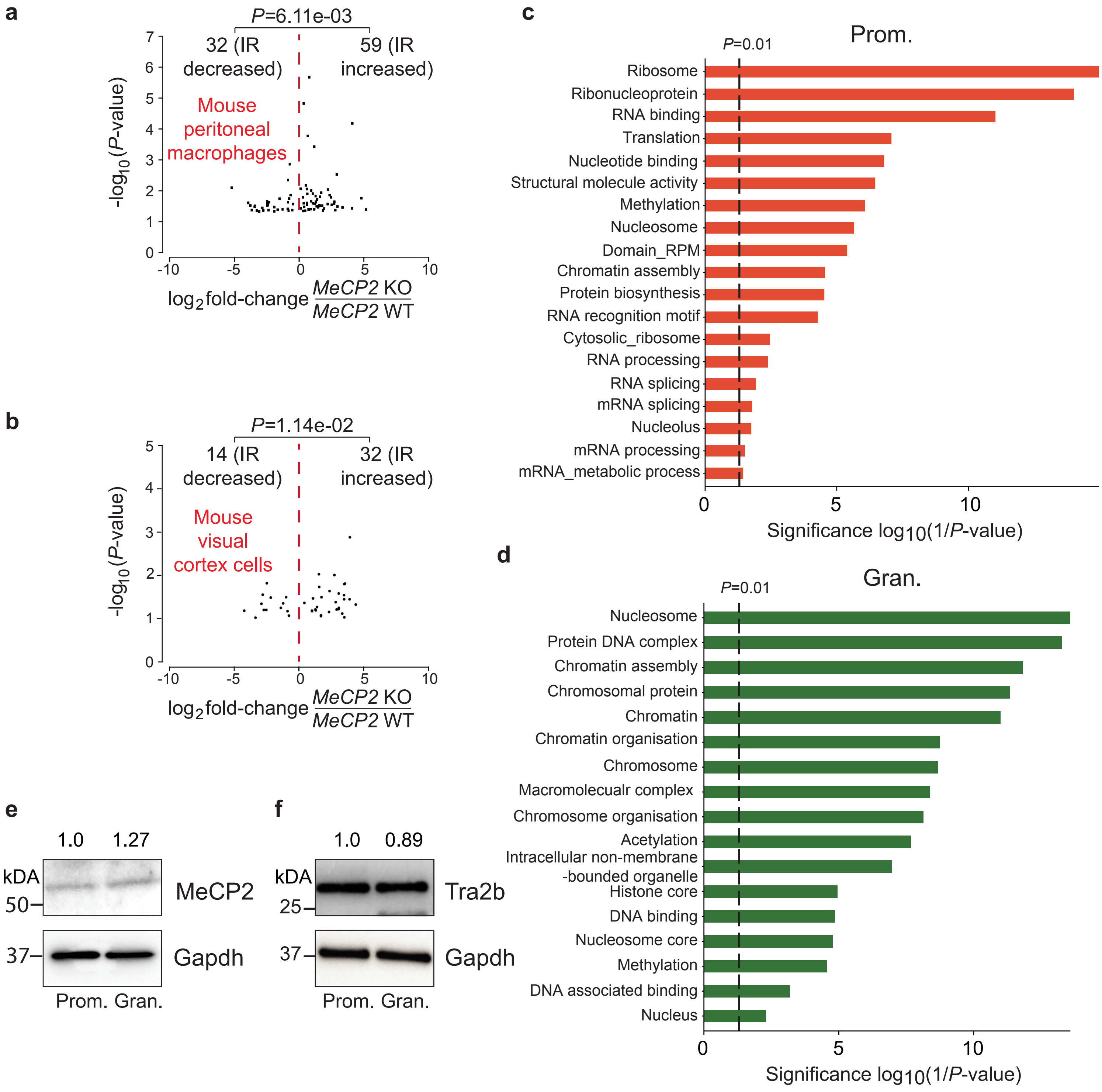

Supplementary Figure 5. MeCP2 levels and occupancy are associated with IR and splicing factor recruitment. Association between IR ratio fold changes and their significance in the MeCP2-knockout primary mouse peritoneal macrophages **(a)** and visual cortex cells **(b)**. Binomial test was used to determine the significant bias between increased and decreased IR at $P<0.05$. Gene ontology analysis of the proteins that interact with MeCP2 in promyelocytes (Prom.) **(c)** and granulocytes (Gran.) **(d)** using RIME ($P<0.01$ with Benjamini-Hochberg correction). **(e and f)** Western blots showing total levels of MeCP2 and Tra2b proteins in promyelocytes and granulocytes. Gapdh was the loading control, with relative levels shown above normalized to Gapdh. Molecular weight markers in kilodalton (kDA) are indicated.

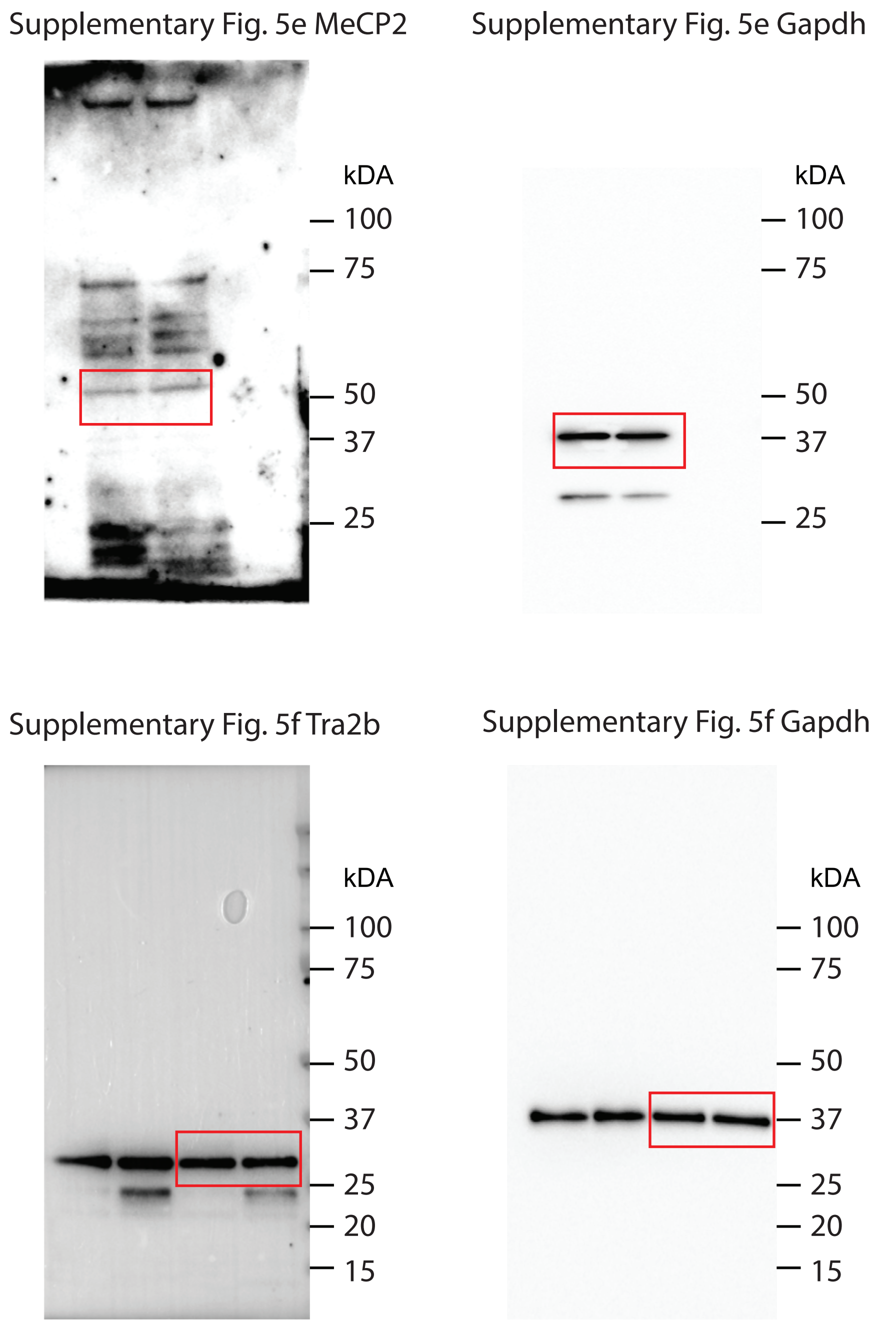

Supplementary Figure 6. Uncropped western blots for cropped images (in red boxes) presented in Supplementary Figures 5e and 5f. Molecular weight markers in kilodalton (kDA) are indicated.

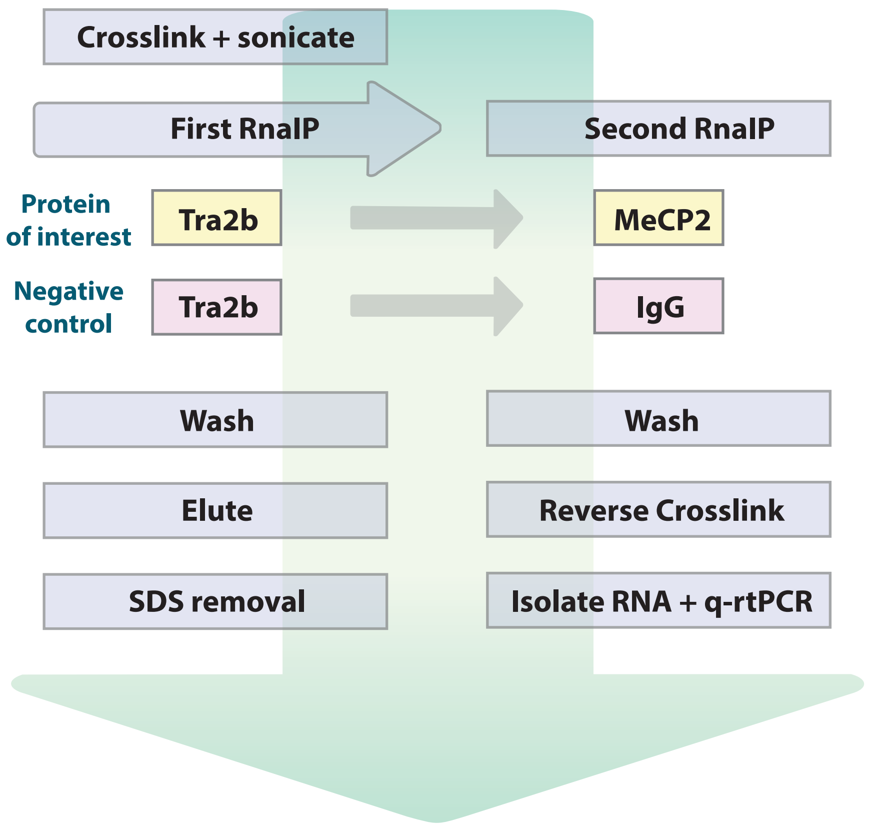

Supplementary Figure 7. Workflow of the RNA-IP-reIP experiment to determine reduced co-occupancies of MeCP2 and Tra2b at splice junctions near retained introns.

Supplementary Table 1. WGBS, ChIP and RNA-seq data analyzed.

**Paired WGBS and RNA-seq**			
**Cell line/Primary cells**	**BS-seq ID**	**RNA-seq ID**	**Organism**
Wildtype fibroblast	GSM1134890	GSM1134903	mouse
Fibroblast reprogrammed for 3 weeks	GSM1134892	GSM1134902	mouse
H1	GSM429321	GSM438361	human
H9	GSM1521762	GSM1521768	human
Neuron progenitor	GSM1521763	GSM1521770	human
IMR90	GSM432687	GSM1151056	human
HCT116	GSM1465024	GSM1151050	human
**RNA-seq of DNMT knockout or 5-Aza treated cells and controls**			
**Cell line/Primary cells**	**Control ID**	**KO/Treated ID**	**Organism**
B cell	GSM1229021	GSM1229013	mouse
Hematopoietic stem cell	GSM1206271	GSM1229019	mouse
OCI-AML3	GSM1329859-61	GSM1329862-64	human
HCT116	GSM1151050	GSM1151051	human
**RNA-seq of MeCP2 knockdown or knockout cells and controls**			
**Cell line/Primary cells**	**Control ID**	**KO/Treated ID**	**Organism**
IMR90	GSM1151048	GSM1151053	human
Peritoneal macrophage	GSM1617050	GSM1617051	mouse
Cerebellum	GSM1063328	GSM1063324	mouse
Visual cortex	GSM1643940-42	GSM1643943-45	mouse

Supplementary Table 2. Proteins that coimmunoprecipitated with MeCP2 in promyelocytes and/or granulocytes as detected by the RIME assay.

Detected in Promyelocytes		
Name	Total number of peptide	References to known MeCP2 interactors
Histone H1.3	40	
Nucleolar RNA helicase 2	30	Huttlin, E. L., et al. (2015). Cell
Histone H3.1	29	Lambert, J. P., et al. (2015). J Proteomics
40S ribosomal protein S8	21	Huttlin, E. L., et al. (2015). Cell
Histone H4	18	
Histone H1.4	15	
60S ribosomal protein L4	15	
Serine/arginine-rich splicing factor 2	13	
U1 small nuclear ribonucleoprotein 70 kDa	13	
Core histone macro-H2A.1	12	Chahrour, M., et al. (2008). Science
Scaffold attachment factor B1	11	
60S ribosomal protein L13	11	Huttlin, E. L., et al. (2015). Cell
40S ribosomal protein S6	10	
60S ribosomal protein L7a	10	Huttlin, E. L., et al. (2015). Cell
60S ribosomal protein L8	10	Huttlin, E. L., et al. (2015). Cell
Nucleolin	10	Maxwell, S. S., et al. (2013). RNA Biol
60S ribosomal protein L7	9	
Serine/arginine-rich splicing factor 6	9	Tsujimura, K., et al. (2015). Cell Rep
60S ribosomal protein L29	9	Huttlin, E. L., et al. (2015). Cell
SAFB-like transcription modulator	9	
60S ribosomal protein L18	9	Huttlin, E. L., et al. (2015). Cell
rRNA 2'-O-methyltransferase fibrillarin	9	
Serine/arginine-rich splicing factor 10	9	
Transformer-2 protein homolog beta	8	
60S ribosomal protein L23a	8	
Peptidyl-prolyl cis-trans isomerase A	8	
Serine/arginine-rich splicing factor 1	7	
Serine/arginine-rich splicing factor 3	7	
RNA-binding protein 39	6	
Echinoderm microtubule-associated protein-like 4	6	
Nucleophosmin	6	Li, R. et al. (2016) Plos Genet
Serine/arginine-rich splicing factor 4	6	Tsujimura, K., et al. (2015). Cell Rep
40S ribosomal protein S24	5	
40S ribosomal protein S11	4	
Putative RNA-binding protein Luc7-like 2	4	
Serine/arginine-rich splicing factor 7	4	Tsujimura, K., et al. (2015). Cell Rep
Transformer-2 protein homolog alpha	4	
RNA-binding protein with serine-rich domain 1	3	
Histone H2B type 2-B	3	
Protein RCC2	3	
Bcl-2-associated transcription factor 1	3	

Detected in Granulocytes		
Name	Total number of peptide	References to known MeCP2 interactors
Histone H1.5	47	
Histone H1.1	26	
Histone H3.1	24	Lambert, J. P., et al. (2015). J Proteomics
Histone H1.4	17	
Histone H4	12	
Protein S100-A9	8	
Histone H1.3	7	
Histone H2A type 1-H	7	
60S ribosomal protein L6	6	
60S ribosomal protein L23a	3	
Histone H2B type 1-C/E/G	3	
Histone H2B type 2-B	3	

Note: Proteins detected in both promyelocytes and granulocytes are highlighted in green
Splicing factors are highlighted in red

Maxwell, S. S., et al. (2013). "Chromatin context and ncRNA highlight targets of MeCP2 in brain." RNA Biol 10(11): 1741-1757.
Tsujimura, K., et al. (2015). "miR-199a Links MeCP2 with mTOR signaling and Its dysregulation leads to Rett Syndrome phenotypes." Cell Rep 12(11): 1887-1901.
Chahrour, M., et al. (2008). "MeCP2, a key contributor to neurological disease, activates and represses transcription." Science 320(5880): 1224-1229.
Huttlin, E. L., et al. (2015). "The BioPlex network: A systematic exploration of the human interactome." Cell 162(2): 425-440.
Lambert, J. P., et al. (2015). "Proximity biotinylation and affinity purification are complementary approaches for the interactome mapping of chromatin-associated protein complexes." J Proteomics 118: 81-94.
Li, R., et al. (2016). "Misregulation of alternative splicing in a mouse model of Rett Syndrome." Plos Genet 12(6): e1006129.

Supplementary Table 3. Primer pairs used.

RT-qPCR Primers to quantify intronic and exonic expression			
**cDNA target**	**Primer**	**Sequence (5'-3')**	**Related Figures**
Lmnb1 intron 5	Forward	CAAGCTTGAGAATGCCAGAC	2c
	Reverse	TGTCTCATCTGGGACAAAAGA	2c
Lmnb1 exons 5-6	Forward	GCCAGACTCTCCTCAGAGATG	2c
	Reverse	AACACGCTCTAGACTCTTTCTGC	2c
Ngp intron 3	Forward	CAGTCCTCCGTGACTCATCA	2d
	Reverse	CCACCATCACAGCAGAGAGA	2d
Ngp exons 3-4	Forward	GACAGGGATTGCAGTCGAGA	2d
	Reverse	CACCTCCCTCCTCTTTCCAG	2d
Spata13 intron 8	Forward	TGAGAGAAGGGACGTGGAGA	2e
	Reverse	GCAGCTGGTACAGCAGAAGG	2e
Spata13 exons 8-9	Forward	TTGCGCAGCTAGCCACTATT	2e
	Reverse	CCTGGGTGGTTGTTGCAGTA	2e
S100a8 intron 2	Forward	GCCCTCCAAGTTGCTTTCTG	2f
	Reverse	ATCGCAAGGAACTCCTCGAA	2f
S100a8 exons 2-3	Forward	AGGAAATCACCATGCCCTCT	2f
	Reverse	CTCCTTGTGGCTGTCTTTGTG	2f
Atf4 intron 2	Forward	CCTGGTGTGCCCTTTTCATA	Supplementary 4g
	Reverse	CTGGGAAAAGAAATTCAGGTG	Supplementary 4g
Atf4 exons 2-3	Forward	GCAAGGAGGATGCCTTTTC	Supplementary 4g
	Reverse	AGAGCTCATCTGGCATGGTT	Supplementary 4g
Mmp8 intron 1	Forward	GCAGGTGCCACTCCTCTAAG	Supplementary 4c
	Reverse	TGGAGGTGACAGAGAGCAGA	Supplementary 4c
Mmp8 exons 1-2	Forward	GCCTTCCCAGTACCTGAACA	Supplementary 4c
	Reverse	AGCGCTGCATCTCTTTAAGC	Supplementary 4c
S100a9 intron 2	Forward	GCAATGTGTGGTGCCCTATT	Supplementary 4d
	Reverse	TGTCTCACCATCCTCCCAAC	Supplementary 4d
S100a9 exons 2-3	Forward	AGGAAGGAAGGACACCCTGA	Supplementary 4d
	Reverse	GTTTGTGTCCAGGTCCTCCA	Supplementary 4d
Gart intron 19	Forward	AGCCGAGGATGTACCACTGT	Supplementary 4e
	Reverse	CACTTAGAGGGTGGCTGAGG	Supplementary 4e
Gart exons 19-20	Forward	TGGTATTCCCACCAGGGTAA	Supplementary 4e
	Reverse	CATGAACCCTGCAAGACAGA	Supplementary 4e
Gadd45a intron 3	Forward	TGCCTGGTTACTTGTTGCTG	Supplementary 4f
	Reverse	AAGACCTCCCTTCCAAGCAT	Supplementary 4f
Gadd45a exons 3-4	Forward	TGGTGACGAACCCACATTCAT	Supplementary 4f
	Reverse	ATTCGGATGCCATCACCGTT	Supplementary 4f

Primers for chromatin immunoprecipitation			
**Target**	**Primer**	**Sequence (5'-3')**	**Related Figures**
*Gadd45a-in3*ex4	Forward	GCAGGATCCTTCCATTGTGA	3b
	Reverse	ACAGAATGTTCGGGGTTTGG	3b
Coro1a-in1ex2	Forward	GTTGCTCTGGGGATGACAAA	3b
	Reverse	ATTTGCTGGAGCGAACCAC	3b
Mmp8-in1ex2	Forward	CGGAGCAATTTCACAGCAAG	3b
	Reverse	GCTTCTCTGCAACCATCGTG	3b
S100a9-in2ex3	Forward	GACACTCCCTGTGCTTCAGA	3b
	Reverse	GTCCTGGTTTGTGTCCAGGT	3b
Gart-in19ex20	Forward	TGCTGATCCTCTGAAAATAGGC	3b
	Reverse	CTTCCAGGACGTGGTCAACT	3b
*Spata13-ex*8in8	Forward	GGATCCTGCTTTCTTGAGCA	3b
	Reverse	GTCTTTGGCGGAGACATCTT	3b
Fth1-in3ex4	Forward	TTGGGAGAGCTGACCAGTAA	3b
	Reverse	CGTGGTCACCCAGTTCTTTA	3b
Mki67-in6ex7	Forward	GCGCCATGTTTCAGTGTCTT	3b
	Reverse	CATAGGCATTCCCTCACTCTTG	3b

Atf4-in2ex3	Forward	TCTGCCTCCCGAATATGACA	3b,c
	Reverse	CCATTCGAAACAGAGCATCG	3b,c
Smarcd1-ex9in9	Forward	TCTCAGCGGATGAAGTTCTCA	3b
	Reverse	GCACACACTCACCTGATGACA	3b
Nomo1-ex24in24	Forward	GAAGGAGTTCCGCTTTGAGC	3b
	Reverse	ACACGCACCTGTAGGCAGTT	3b
Pam-ex2in2	Forward	GCCTTCAGAAGCCCACTTTC	3b
	Reverse	CAACGTCCTTCCTCCAGTCT	3b
Xist-CpG	Forward	CAGCGCAATTGGTTGCTTT	3b
	Reverse	CCATTATGGCTTCTGCGTGA	3b
IAP	Forward	GCTTTCGTTTTTGGGGCTTGG	3b
	Reverse	CTTACTCCGCGTTCTCACGAC	3b
Gapdh-CpG	Forward	GTCTCTGGAACAGGGAGGAG	3b
	Reverse	CCCTTGAGCTAGGACTGGAT	3b
Actb-CpG	Forward	GGAATGTGGCTGCAAAGAGT	3b
	Reverse	ATCACTCAGAACGGACACCA	3b
Lmnb1-in4 (P1)	Forward	TACCAGAAGGGCTTGGGTTT	4a,b
	Reverse	GGAAGCCACTGTCAGCCTTA	4a,b
Lmnb1-ex5in5 (P2)	Forward	AGCCGGATGAGGATCGAGAG	3b,c and 4a,b
	Reverse	AGCCTTCCAGCTACACCTCA	3b,c and 4a,b
Lmnb1-in5-1 (P3)	Forward	TGGTGAGGTGCCTTCTTTCT	4a,b
	Reverse	GTCACTTGTCGCTGGGGTAT	4a,b
Lmnb1-in5-2 (P4)	Forward	TCAGGAGCTTGGAATGGCTA	4a,b
	Reverse	TGCACTGTATTTGCCCTCCT	4a,b
Lmnb1-in5-3 (P5)	Forward	CTGAGGGTTGTTGGGTCACT	4a,b
	Reverse	GCATAGGTCACACTGGCAGA	4a,b
Lmnb1-in5-5 (P6)	Forward	CTCGGCTTCTGGACCTTCTC	4a,b
	Reverse	GCACGTTCATGTGTGCAATC	4a,b
Lmnb1-in5-6 (P7)	Forward	CCGATTGAGACTGGTGCATT	4a,b
	Reverse	CGCCAGCATATTCCTTCTCA	4a,b
Lmnb1-in5ex6 (P8)	Forward	GTGCTCCTTTGACCCTGAAG	4a,b
	Reverse	CTGCATCTGGTCCCTGATCT	4a,b
Ngp-ex3in3 (P1)	Forward	AACCTCCCTGACCTTGACCT	4a,b
	Reverse	AGAGGCCTGGAGTGGACAT	4a,b
Ngp-in3-1 (P2)	Forward	TGGGAGACACTGAGTTGCTG	4a,b
	Reverse	TGACAAAGGACATGCCACAC	4a,b
Ngp-in3-2 (P3)	Forward	TTCCCACTGTTAGGGGAGTG	4a,b
	Reverse	AGGCTTCCATAGGCTGTCAT	4a,b
Ngp-in3-3 (P4)	Forward	CCCCAATTCAGATCTGCTCA	4a,b
	Reverse	CAGCTACAAGGGCATGATGA	4a,b
Ngp-in3ex4 (P5)	Forward	CTGCTGTGATGGTGGTGACT	3b,c and 4a,b
	Reverse	ATGTGAGGGGGCACATCTT	3b,c and 4a,b
S100a8-ex2in2 (P1)	Forward	AGGAAATCACCATGCCCTCT	4a,b
	Reverse	CCATCCCAGCACCATTAGAA	4a,b
S100a8-in2 (P2)	Forward	TTCTAATGGTGCTGGGATGG	4a,b
	Reverse	AAGCGAGGACTATCTGTGGA	4a,b
S100a8-in2ex3 (P3)	Forward	TCCACAGATAGTCCTCGCTTA	3b and 4a,b
	Reverse	ATCGCAAGGAACTCCTCGAA	3b and 4a,b
Lmnb1-ex3in3 (P1)	Forward	GGATTTGGAGAATCGCTGTC	4c,d
	Reverse	GGCCAGGACTATGCAGAGAA	4c,d
Lmnb1-in3-1 (P2)	Forward	CTCTGTAACAAAGCCGCACA	4c,d
	Reverse	CTGCAAGGCATCTCCTATCC	4c,d
Lmnb1-in3-2 (P3)	Forward	AGACCCTGCCTGCATACATC	4c,d
	Reverse	CAATCCCTACGGACACCTCT	4c,d
Lmnb1-in3ex4 (P4)	Forward	TTGAGTCAGGACAGGCACAG	4c,d
	Reverse	GGTCTCATGCTTCCTCCTTG	4c,d
Smarcd1-ex9in9 (P1)	Forward	TCTCAGCGGATGAAGTTCTCA	4c,d
	Reverse	GCACACACTCACCTGATGACA	4c,d
Smarcd1-in9-1 (P2)	Forward	ACCAACTTGTGCTTGCCTGT	4c,d
	Reverse	TAGCATGGGCCAGAGAAGAG	4c,d

Smarcd1-in9-2 (P3)	Forward	AGCAAGATGGTTCAGCAGGT	4c,d
	Reverse	GGTGAGAGGACCATTTGTGG	4c,d
Smarcd1-in9ex10 (P4)	Forward	TCATCCCTGGACCATCTTTC	4c,d
	Reverse	CAGAGTGTCATCCACCTCCA	4c,d
TBP-ex5in5 (P1)	Forward	AAGAGAGCCACGGACAACTG	4c,d
	Reverse	CCCACTAGAAACAAAGCATTCC	4c,d
TBP-in5-1 (P2)	Forward	TGGTTTGCTCTGATTACTCTGC	4c,d
	Reverse	AATGGCAGTGCTACAACCAG	4c,d
TBP-in5-2 (P3)	Forward	AGGTCATAATGAGGTGATGACG	4c,d
	Reverse	TGTGGCACATTACTGTCAAGC	4c,d
TBP-in5ex6 (P4)	Forward	TGGTTTCTGTTGAGGACACG	4c,d
	Reverse	TTCTTGCTGCTAGTCTGGATTG	4c,d
Chr6-intergenic	Forward	TACCAATGTCCACCCTCTGA	4b,d
	Reverse	GACAACATCCACACGTCCAG	4b,d

**Primers for bisulfite sequencing**			
**Target**	**Primer**	**Sequence (5'-3')**	**Related Figures**
Lmnb1-in4ex5in5	Forward	TTAGTTGGTTATTAGAAGGGTTTGG	1d and 2c
	Reverse	AAAAAAATAACACCAAAAAAACAAC	1d and 2c
Ngp-in3ex4	Forward	ATTTTGGGGTTTGGGAATATTATAT	1d and 2d
	Reverse	TATACAAAAAACACCTCCCTCCTC	1d and 2d
S100a8-in2ex3	Forward	TTTGTGTAGGTGAGGAGGTGTT	1d and 2f
	Reverse	AACCCAACCCTAAACCAAAAA	1d and 2f
Spata13-ex8in8	Forward	AAATTGTTTAATTGTTTTTAAGTAAATAAA	2e
	Reverse	ACAACACCCTCTAAAATATCTTTAAC	2e
Atf4-in2ex3	Forward	TATGGATGATGGTTTGGTTAGTGT	Supplementary 4g
	Reverse	CAAAATCAAACTTCCTATCTCCTTC	Supplementary 4g
MMp8-in1ex2	Forward	AATTTTTGTTATTGTTTTTTGTTTGT	Supplementary 4c
	Reverse	CCAAAATCAAACACTCCACATC	Supplementary 4c
S100a9-in2ex3	Forward	AGTATTGTGTTTTAAATTAAATTTAGATTT	Supplementary 4d
	Reverse	TTACCATAACTATAACCATACCCAC	Supplementary 4d
Gart-in19ex20	Forward	TTGGGTTTGTTTTTTTGTTGTATTT	Supplementary 4e
	Reverse	TCAATCAATCTAAACACTTCCTACC	Supplementary 4e
Gadd45a-in3ex4	Forward	ATTTATTAGGGTATATGTTTGGAAGG	Supplementary 4f
	Reverse	ACAATTTAATTCAATTATTTCCATTC	Supplementary 4f

**Primers for RNA-IP and RNA-IP-reIP**			
**Target**	**Primer**	**Sequence (5'-3')**	**Related Figures**
Malat1	Forward	GTTACCAGCCCAAACCTCAA	5c
	Reverse	CACTTGTGGGGAGACCTTGT	5c
Tra2a	Forward	GAATTGGGGAAGAATACACGAA	5d
	Reverse	AGGACCCATTCATTCTTCCAG	5d
Lmnb1	Forward	GAGAATGCCAGACTCTCCTCA	5e,f,i
	Reverse	GAGGCTCTCGATCCTCATCC	5e,f.i
Ngp	Forward	GACAGGGATTGCAGTCGAGA	5e,f,i
	Reverse	CACCTCCCTCCTCTTTCCAG	5e,f,i
S100a8	Forward	TCGTGACAATGCCGTCTGAA	5e,f,i
	Reverse	AGGGCATGGTGATTTCCTTGT	5e,f,i
Smarcd1	Forward	TCTCAGAGATCCCTCAGCGG	5g,h,i
	Reverse	CCACCTCCACGTCAATGTCA	5g,h,i
Tbp	Forward	AAGAGAGCCACGGACAACTG	5g,h,i
	Reverse	GCTCCTGTGCACACCATTTT	5g,h,i
